# From vineyard to genome: optimized enrichment and sequencing of Flavescence dorée phytoplasma from grapevine samples

**DOI:** 10.1099/mgen.0.001514

**Published:** 2025-09-29

**Authors:** Zala Kogej Zwitter, Denis Kutnjak, Natasa Mehle

**Affiliations:** 1 Department of Biotechnology and Systems Biology, National Institute of Biology, Ljubljana, Slovenia; 2 Jožef Stefan International Postgraduate School, Ljubljana, Slovenia; 3 School for Viticulture and Enology, University of Nova Gorica, Nova Gorica, Slovenia

**Keywords:** enrichment, grapevine, HTS, nanopore, plant pathogen

## Abstract

Phytoplasmas are non-culturable obligate intracellular bacteria that cause considerable economic losses in agriculture. Genome sequencing provides crucial insights into their biology and vector dependence. However, genome studies on phytoplasmas are often hampered by their low abundance in naturally infected plants. Propagation in test plants is usually necessary but time-consuming and resource-intensive, especially for quarantine phytoplasmas such as the phytoplasma causing Flavescence dorée (FD), a serious threat to European viticulture. To overcome these challenges, we aimed to develop a protocol for efficient enrichment of phytoplasma DNA directly from field-collected samples, enabling genome sequencing using both Illumina and Oxford Nanopore Technologies platforms. We evaluated six sample preparation protocols that included stepwise enrichment steps to improve phytoplasma genome coverage and assembly quality. The most effective approach combined differential centrifugation, CTAB extraction and removal of CpG-methylated host DNA and resulted in a notable increase in the relative abundance of phytoplasma reads compared to other protocols. Rarefaction analysis of the dataset generated using this protocol demonstrated that the entire phytoplasma genome was covered by reads in a dataset comprising 3 billion nucleotides. We also evaluated and compared *de novo* phytoplasma genome assemblies generated from short Illumina reads and long nanopore sequencing reads. While Illumina sequencing yielded more accurate assemblies with longer total lengths, the assemblies derived from nanopore sequencing data contained longer individual contigs. This advantage was reflected in hybrid assemblies that combined both technologies, yielding longer phytoplasma contigs than assemblies from Illumina datasets and lower mismatch rates compared to assemblies from nanopore sequencing datasets. A hybrid *de novo* assembled genome of the Slovenian FD phytoplasma isolate achieved 96% reference genome coverage, with high contiguity and low error rates. This streamlined and accessible protocol enables high-quality genome sequencing of phytoplasma-infected grapevines without the need for propagation in test plants. This facilitates broader phytoplasma research and can potentially be extended to other naturally infected phytoplasma hosts or organisms infected with other non-culturable microbes.

**Impact Statement**

Flavescence dorée (FD) disease is a major threat to European viticulture. It is associated with a quarantine phytoplasma that is difficult to analyse due to its low abundance in infected plants and the high proportion of host DNA in samples. Current methods for sequencing phytoplasma genomes often require propagation in test plants, a time-consuming and resource-intensive process. In this study, we developed an accessible, field sample-based protocol for phytoplasma DNA enrichment that eliminates the need for specialized facilities such as quarantine greenhouses. By optimizing a combination of pre- and post-extraction enrichment steps, we achieved high genome coverage and high-quality assembly of a Slovenian FD phytoplasma isolate directly from infected grapevines using high-throughput sequencing. This approach simplifies phytoplasma genomics research and enables more efficient monitoring, epidemiological studies and development of diagnostic tools. In addition, this protocol could be adapted for sequencing other naturally infected phytoplasma hosts and plants infected with other non-culturable bacteria, expanding its potential applications in plant pathology and microbial genomics.

## Data Summary

The developed protocol is accessible at DOI: dx.doi.org/10.17504/protocols.io.8epv52w5jv1b/v1. All genome sequencing data, including sequencing reads (SRX28207723, SRX28087407, SRX28087406, SRX28087405, SRX28087404 and SRX28087403) and the final genome assembly, are available in the Sequence Read Archive and NCBI genome repositories under the BioProject number PRJNA1231790 and GenBank number GCA_050897995. The authors confirm that all supporting data, codes and protocols have been provided in this article or in supplementary data files.

## Introduction

Phytoplasmas are obligate intracellular phytopathogenic bacteria that infect more than 1,000 plant species worldwide [1]. By disrupting the growth of stem, leaf or fruit tissue, phytoplasmas reduce the yield or survival of infected plants, including commercially valuable crops, ornamentals and fruit trees [2]. These non-culturable bacteria belong to the class *Mollicutes* and the taxon ‘*Candidatus* Phytoplasma’, which is categorized into groups, subgroups and species based on the genetic information of 16S rRNA and other conserved genes [3,4]. Phytoplasmas have no cell wall and reproduce in the phloem of plants and in the haemolymph of insect vectors [5]. Insects that feed on the phloem sap of infected plants, such as leafhoppers, planthoppers and psyllids, transmit phytoplasmas from one plant to another [6]. The host range of phytoplasmas is therefore determined by the feeding preferences of these vectors [7]. Phytoplasmas can also spread by vegetative propagation methods such as cuttings, grafts and micropropagation [1].

As they cannot grow in cell-free media, molecular methods targeting DNA sequences are essential for the detection and investigation of phytoplasmas. Genome sequencing is particularly valuable for understanding their biology, host dependence and interactions with insect vectors and plants [8]. However, sequencing of complete phytoplasma genomes remains a major challenge, as phytoplasma DNA typically comprises less than 1% of the total nucleic acids extracted from infected plant material [9]. This is not only due to their smaller genome size compared to their hosts [10], but also to their generally low concentrations in natural hosts, which can vary considerably depending on the plant organ [11,12] and growth stage [13]. The high proportion of host DNA complicates sequencing and increases costs, while the high AT content (often more than 70%) and the numerous duplicated genes in phytoplasma genomes cause difficulties in assembly based on short-read sequencing [14].

To overcome these challenges, many studies propagate phytoplasmas in alternative hosts, such as *Catharanthus roseus* or *Vicia faba*, by transferring them to these hosts through grafting [15], dodder (*Cuscuta* sp.) [16] or insect vectors [17]. These test plants often harbour higher phytoplasma concentrations [18], making them suitable starting material for genome sequencing. Propagation in test plants is time-consuming and requires specialized, pest-proof greenhouses, especially for quarantine pests such as Flavescence dorée phytoplasma (FDp). Unfortunately, there is a lack of protocols that would allow direct sequencing of field samples, especially of woody plants. Enrichment methods can help solve these problems by increasing the phytoplasma DNA concentration after total DNA extraction. Common techniques include PFGE [15,16, 19,21] and caesium chloride buoyant-density gradient centrifugation [16,22]. Non-toxic alternatives such as iodixanol gradients have also been used for DNA separation [23]. Other enrichment approaches include immunoprecipitation-based methods [24], methyl-CpG binding domain-mediated techniques [25] and DNA amplification strategies such as multiple displacement amplification [26] and rolling circle amplification. Methods such as subtractive hybridization and mirror orientation selection have also been shown to be effective for the enrichment of phytoplasma DNA [9].

As of 10 July 2025, the NCBI Genome database lists 38 complete genomes of 16 different phytoplasma species, generated using different enrichment methods and sequencing platforms [15,17,19]. While short-read platforms such as Illumina are commonly used, long-read platforms such as Oxford Nanopore Technologies (ONT) and PacBio are becoming increasingly popular. These platforms can be combined in hybrid assemblies to improve the quality of the genome [43]. Nanopore sequencing offers advantages for *de novo* assembly as it can generate long reads without the need for DNA amplification or synthesis during the sequencing process. However, the achievement of high-quality long reads is highly dependent on sample and library preparation, especially the extraction and handling of intact, high-molecular-weight DNA [44].

Nanopore sequencing in combination with Illumina sequencing data was used to complete the first and only available genome sequence of FDp propagated in test plants [17]. FDp from taxonomic subgroups 16SrV-C and 16SrV-D [45] is a quarantine pest in the EU (Regulation 2019/2072) and the causal agent of a serious grapevine disease that is widespread in European vineyards [46]. First documented in France in the 1950s [47], the spread of FDp has increased rapidly, fuelled by its vector, the invasive species *Scaphoideus titanus* Ball (Hemiptera: Cicadellidae), originally from North America [48]. Despite control measures, the disease persists, emphasizing the need for further research.

To overcome the challenges in obtaining the FDp genome sequence, we have developed an efficient and easily accessible protocol for the direct preparation of field-collected grapevine samples for whole-genome sequencing of phytoplasmas that eliminates the need for propagation in test plants. This protocol integrates and optimizes existing enrichment techniques both pre- and post-DNA extraction to maximize the yield of phytoplasma DNA. The protocol has also been adapted for use with the ONT MinION sequencer, a platform suitable for small laboratories. Different combinations of enrichment methods were evaluated based on their impact on reference genome coverage, sequencing depth and quality of the *de novo* assembled genome. By integrating Illumina and nanopore sequencing data, we successfully generated a high-quality draft genome of a Slovenian FDp isolate, demonstrating the effectiveness and applicability of the protocol for phytoplasma genomics.

## Methods

### Plant material and homogenization

The plant material used in this study was a symptomatic grapevine sample from a vineyard in the northeast of Slovenia, variety 'Renski rizling', collected in the second half of August 2020 (sample ID D933/20). The sample consisted of leaves taken from different parts of the plant. The leaf veins were cut out and chopped into small pieces with a sterile scalpel. To ensure that the sampled grapevine was infected with FDp, DNA was extracted from 1 g of leaf veins using the QuickPick^™^ SML Plant DNA kit (Bio-Nobile) and the KingFisher mL Purification System (Thermo Fisher Scientific) [49]. The DNA was then subjected to nested PCR and Sanger sequencing of the secY-map locus with FD9f5/MAPr1 and FD9f6/MAPr2 primer pairs [50]. Determination of the *map* genotype and conformation of infection with FDp was performed according to the procedure described by Kogej Zwitter *et al*. [51]. Subsequently, a total of 10 g of small pieces of leaf veins was homogenized with a mortar and pestle using liquid nitrogen. Using a pre-cooled spatula, the powdered plant tissue sample was transferred into pre-cooled 15 ml tubes. Six aliquots containing 1 g of homogenized plant tissue were prepared from this sample. The tubes containing the plant material were then immediately stored at −80 °C.

To assess the consistency of the enrichment, two additional samples of grapevine and two samples of hazelnut were prepared and homogenized using the same procedure as described above. The grapevine samples were collected in northeast Slovenia – one at the end of August 2020 (sample ID: D1062/20, unknown cultivar) and the other at the beginning of June 2025 (sample ID: 572/25, cultivar Pinot Gris). Both hazelnut samples came from phytoplasma-infected orchards in the same region. One was collected in mid-July 2021 (sample ID: BS21-P-1) and the other in mid-October 2020 (sample ID: 1461/20). The latter differed from the others in that it originated from root phloem tissue and not from leaf veins.

### Comparison of different protocols for phytoplasma sequencing

**Fig. 1.**
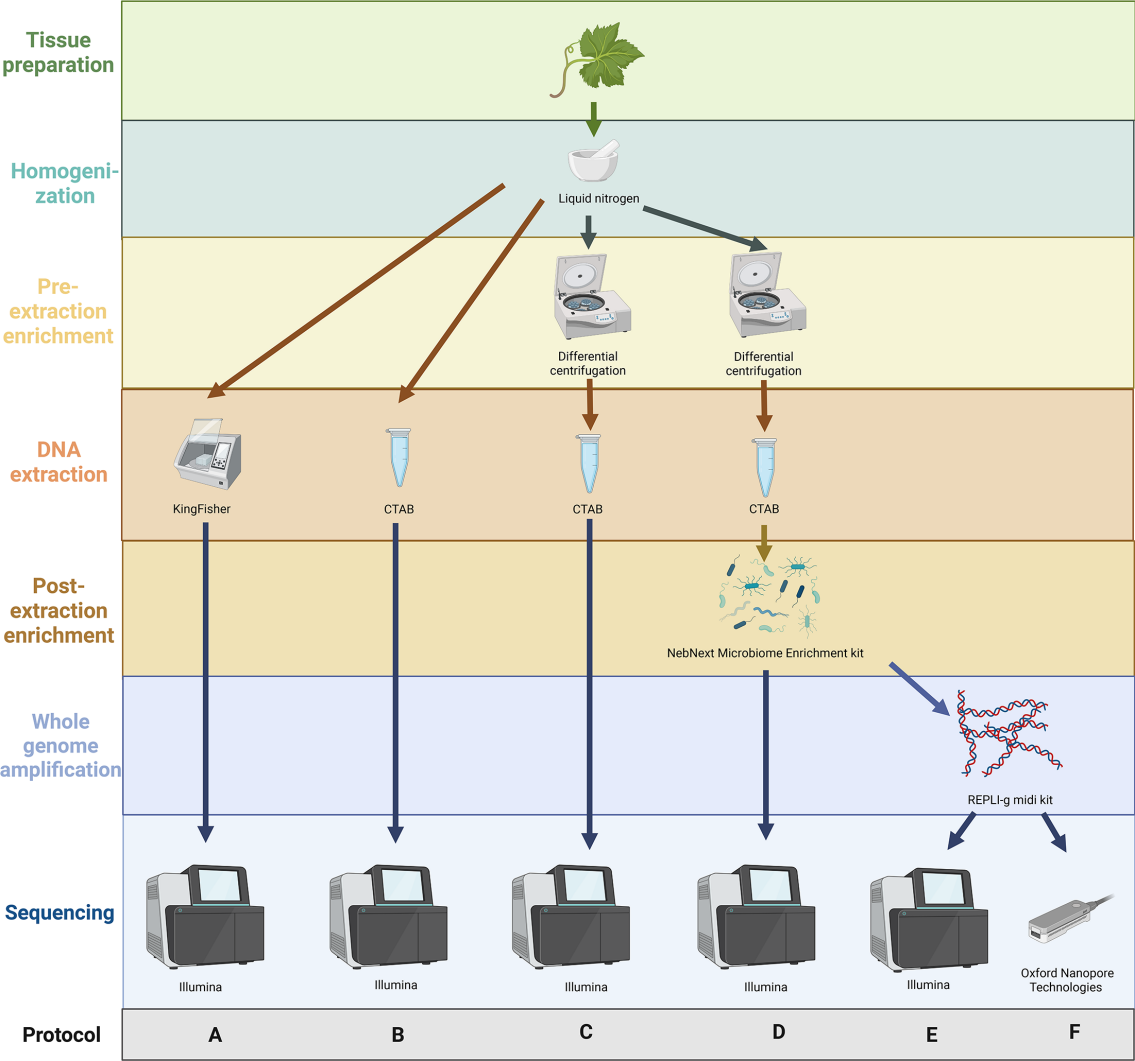
Schematic representation of the workflow and testing of different protocols (A–F) used to obtain the genomic sequence of FDp. All protocols started with a homogenized sample of grapevine leaves (D933/20) crushed in liquid nitrogen (top). The diagram outlines the steps involved in each of the six protocols, labelled A–F at the bottom of the figure. Arrows illustrate the flow of the different steps, which are explained on the left-hand side of the figure, with additional information next to each step.

To find out how phytoplasmas can be reliably and rapidly enriched and their genomic sequences obtained from a field grapevine sample (D933/20), aliquots of the homogenized sample were analysed using six different protocols (named as protocols A–F). In these protocols, one step was added at a time, so that we could assess the impact of each step on the final result. The steps for each protocol are listed below and shown schematically in Fig. 1, with detailed procedures described in the following sections:

–A: DNA extraction with KingFisher (ThermoScientific, USA) and Illumina sequencing
–B: DNA extraction with CTAB and Illumina sequencing
–C: differential centrifugation, DNA extraction with CTAB and Illumina sequencing
–D: differential centrifugation, DNA extraction with CTAB, enrichment with NebNext Microbiome DNA Enrichment Kit (New England Biolabs, MA, USA) and Illumina sequencing
–E: differential centrifugation, DNA extraction with CTAB, enrichment with NebNext Microbiome DNA Enrichment Kit (New England Biolabs, MA, USA), whole-genome amplification (WGA) and Illumina sequencing
–F: differential centrifugation, DNA extraction with CTAB, enrichment with NebNext Microbiome DNA Enrichment Kit (New England Biolabs, MA, USA), WGA and ONT sequencing.

The four additional samples of grapevine and hazelnut (D1062/20, 572/25, BS21-P-1, 1461/20) were prepared according to protocols A, C and D up to the sequencing step (Fig. 1).

### Pre-extraction enrichment: differential centrifugation

The first step in the enrichment of phytoplasmas was differential centrifugation. 1 g of the homogenised sample was processed with 10 ml of Phytoplasma Grinding Buffer (PGB: 96 mM K_2_HPO_4_, 30 mM KH_2_PO_4_, 10% sucrose, 0.15% bovine serum albumin, 2% polyvinylpyrrolidone-10, 30 mM ascorbic acid, pH 7.6). The mixture was kept on ice for 10 min to allow the cells to plasmolyse. The extract was then poured into fresh, chilled 15 ml tubes and centrifuged (Sigma, 3-18KS, rotor 12171) at 2,500 ***g*** for 5 min at 4 °C, allowing environmental and cell debris to settle to the bottom. The supernatant was poured into fresh, chilled 15 ml tubes and equilibrated in pairs with PGB. These tubes were then centrifuged at 18,000 ***g*** for 25 min at 4 °C (Sigma, 3-18KS, rotor 12171). The supernatant was then poured off, and the resulting pellet with enriched phytoplasma material was immediately processed using the CTAB extraction method.

### DNA extraction

#### KingFisher total DNA extraction

Semi-automated extraction of total DNA with magnetic particles using the KingFisher device was included in one of the protocols, as it is a rapid extraction option that can be used when processing a large number of samples. To the homogenized plant material, 2 ml of Plant DNA lysis buffer (QuickPick Plant DNA Kit; Bio-Nobile, Finland) was added and vortexed thoroughly for 1 min. The following steps of the extraction protocol using the QuickPick^™^ SML Plant DNA Kit (Bio-Nobile, Finland) and a KingFisher extraction device (ThermoScientific, USA) followed the procedure described in [49].

#### CTAB extraction of total DNA

The CTAB extraction protocol (adapted from [52,53]) was included in five of the compared protocols. In 15 ml tubes, 3 ml of CTAB buffer (4% CTAB, 1% PVP40, 0.2 M Tris-HCl, 1.4 M NaCl, 20 mM EDTA, pH 8) heated to 65 °C was added to homogenized plant material (protocol B), and 2 ml of CTAB buffer with the same composition was added to the pellet after differential centrifugation (protocols C–F). The material was resuspended by vortexing. The tubes were incubated at 65 °C for 1 h, vortexed in between (every 20 min) and then centrifuged at 5,000 ***g*** for 5 min at room temperature. From this step onwards, the same sample was split and processed in two parallel extractions: twice, 1 ml of the sample was transferred to a 2 ml tube, and the same amount of chloroform:isoamyl alcohol (24 : 1) was added to each tube and vortexed. After centrifugation (10 min, 6,000 ***g***, room temperature), the upper aqueous phases were transferred to fresh 1.5 ml tubes. An equal volume of ice-cold isopropanol was added to each tube and mixed by inverting. The DNA was then precipitated for 20 min at room temperature. After centrifugation (15 min, 15,000 ***g***, 4 °C), the pellets were washed with ice-cold 70% ethanol and, after additional centrifugation (2 min, 10,000 ***g***, 4 °C) and drying, resuspended in 50 µl of nuclease-free water. The DNA extracted from two parallel extractions was combined in one tube.

#### Post-extraction enrichment: microbiome enrichment

For enrichment after DNA extraction, the NEBNext^®^ Microbiome DNA Enrichment Kit was used (New England Biolabs, MA, USA), which removes methylated eukaryotic host DNA from the sample. The manufacturer’s protocol was followed, using the maximum amount of DNA possible (up to 1 µg, in this case from 1,050 to 382 ng for different samples in the corresponding sample volume). The collected enriched microbial DNA was cleaned using Agencourt AMPure XP beads (option A in the NebNext Microbiome DNA enrichment protocol) (Beckman Coulter, Brea, CA, USA).

#### Whole-genome amplification

Since the selected nanopore sequencing library requires high amounts of input nucleic acids, that could not be obtained from our sample, an additional WGA step was performed in two of the protocols (E and F) as suggested in the ONT Ligation sequencing gDNA – WGA protocol (SQK-LSK110, version WAL_9115_v110_revH_10Nov2020). To obtain a sufficiently high concentration of total DNA suitable for nanopore sequencing (protocol F and, for comparison, protocol E, using Illumina sequencing), phytoplasma-enriched DNA was subjected to WGA using the REPLIg-midi Kit (Qiagen, Hilden, Germany) with an input of 10 ng of enriched DNA according to the Protocol for Amplification of Purified Genomic DNA using the REPLI-g Midi Kit.

#### Sequencing

For protocols A–E, Illumina sequencing was performed by Novogene (Cambridge, UK) using the Plant and Animal Whole Genome sequencing service on Illumina NovaSeq (PE150 mode) aiming for 5 G bases of raw data per sample.

Nanopore sequencing was used in this study as an option for rapid in-house sequencing and to test whether potentially longer sequencing reads could be beneficial for genome assembly. Nanopore sequencing was performed using the SQL-LSK110 Ligation sequencing kit (Oxford Nanopore Technologies Limited, Oxford, UK). Library preparation followed the Oxford Nanopore protocol Ligation sequencing gDNA – WGA (version: WAL_9115_v110_revH_10Nov2020, Oxford Nanopore Technologies Limited) with some modifications: Mag-Bind Total Pure NGS beads were used for the purification steps (Omega Bio-tek, Norcross, Georgia), and after the endonuclease reaction, cleaning was performed with 1.8 volumes of Mag-Bind Total Pure NGS beads (instead of Custom buffer). In the DNA repair and end-prep step, 1 µl of DNA CS from the SQL-LSK110 kit was added along with 47 µl of prepared genomic DNA (in this case, 1269 ng) as a control of the sequencing run. Long Fragment Buffer was used in the final clean-up step after adapter ligation. Thirty-eight femtomoles of the finally prepared library were loaded onto the flow cell. Sequencing on the MinION with flow cell (R9.4.1) was run for 72 h, with basecalling switched off and a minimum of 20 bp read length.

#### Measurements and quality control of DNA in each protocol

To determine DNA quantity across different protocols, DNA concentrations were measured using the dsDNA HS kit on a Qubit fluorometer (Thermo Fisher Scientific, MA, USA) and with the ND-1000 spectrophotometer (NanoDrop Technologies, Wilmington, USA). For the whole-genome-amplified sample prepared for nanopore sequencing (protocol F), DNA concentration and fragment lengths were determined using the TapeStation and Genomic DNA ScreenTape Kit (Agilent Technologies, Santa Clara, USA) to ensure the presence of high molecular-weight DNA suitable for library preparation and accurate loading of the library onto the flow cell.

Real-time PCR (qPCR) was used to verify the presence of phytoplasma DNA, as well as its host eukaryotic DNA in the samples prepared with each of the protocols prior to sequencing and to evaluate the consistency of the enrichment between samples. Tenfold dilutions of the isolated DNA (added amounts of sample D933/20 DNA ranged between 0.4 and 77.2 ng depending on the protocol, as indicated in Table 1) were tested using the 18S rRNA assay (Applied Biosystems, MA, USA) to control extraction and estimate the level of eukaryotic host DNA, and the specific assay for the 16SrV phytoplasma group [54] in two replicates. The qPCR reactions were performed as described in [55]. In addition to the samples, negative controls of extraction (with water instead of a biological sample) and negative controls of amplification were also tested in two replicates.

**Table 1.** Results of specific tests and bioinformatic analyses on grapevine sample D933/20 prepared with different protocols (A–F, see Fig. 1); DNA yield measured after isolation and enrichment (when performed); mean Cq values of two technical replicates for 18S rRNA and specific 16SrV real-time PCR assay; total HTS dataset size in billion nucleotides, which was subsampled in five replicates to the smallest dataset size for comparison; HTS read mapping results represent the average of five replicates, each subsampled to 4.8 billion nucleotides and mapped to the host (*Vitis vinifera*, GCF_000003745.3) and FDp reference genome (CP097583); consensus sequence length corresponds to the mapping-based consensus

Protocol	A	B	C	D	E	F
DNA concentration (ng μl^−1^)	3.9	28.0	2.2	2.4	386.0	
Total DNA yield (ng)	784	2,800	216	118	11,580	
18S rRNA assay (Cq)	19.1	17.3	21.7	26.0	19.4	
16SrV-specific assay (Cq)	28.9	25.0	28.3	27.3	18.9	
Total nt (billion)	6.1	4.8	4.9	6.0	7.0	7.4
Subsampled total nt (billion)	4.8	4.8	4.8	4.8	4.8	4.8
Nt mapped to host (%)	95.84	96.02	93.89	89.53	91.27	92.48
Nt mapped to FD ref (%)	0.03	0.04	0.07	0.26	0.17	0.15
Consensus length (FDp)	547,983	501,505	576,564	653,574	650,919	653,544
Fraction of FDp ref covered	0.84	0.77	0.88	1.00	1.00	1.00
Average depth (FDp)	3	3	5	19	12	10

Cq, quantification cycle; nt, nucleotides; ref, reference.

#### Bioinformatic analyses

All bioinformatic analyses were performed on the grapevine sample D933/20 using two high-performance servers (for characteristics, see Material S1, available in the online Supplementary Material, and Table 1). The software tools were run via command line, and CLC Genomic Workbench (Qiagen, Hilden, Germany) was running on Server 1.

#### Read quality control, subsampling and mapping to the reference

The short Illumina reads were analysed using CLC Genomic Workbench V23 (Qiagen, Hilden, Germany). The reads were first trimmed of adapters, and the quality limit was set to 0.05 without trimming homopolymers. The sequencing runs resulted in different output sizes. In order to compare the datasets, they were therefore normalized to the same size. The read datasets from the different protocols were randomly subsampled to 4.8 billion nucleotides using the CLC tool Subsample Sequence List, which corresponded to the size of the smallest dataset. Reads were mapped to the *V. vinifera* genome available at NCBI (RefSeq assembly GCF_000003745.3) to evaluate the proportion of reads mapped to the host and to the FDp reference genome sequence CP097583 from GenBank [17], using the parameters specified in Material S1.

The nanopore sequencing reads were first basecalled with Guppy (version 6.4.2). All passed files were merged into one fastq file, and based on the NanoPlot results, reads were trimmed by 50 nucleotides at the head and tail, and reads shorter than 50 nucleotides were discarded, all using NanoFilt. The statistics of the filtered reads were generated using nanoQC, NanoStat and NanoPlot. The merged filtered data were imported into CLC Genomics Workbench (version 24) and analysed using the Long Read Support plugin. The dataset was randomly subsampled to 4.8 billion nucleotides using the CLC Subsample Sequence List tool, estimating the number of reads based on the average read length after NanoFilt filtering. The reads were then mapped to the host (grapevine) and the FDp reference (as indicated above) using the Map Long Reads to Reference tool using the parameters stated in Material S1.

To investigate how the size of the datasets affects the genome coverage and to see how much data is needed to cover the majority of the FDp reference genome by read mapping, all 6 originally obtained datasets were downsized to 0.9, 1.8, 2.4, 3, 3.6 and 4.8 billion nucleotides in 5 replicates as stated above. Reads from each replicate were mapped to the FDp reference genome as described above. Data visualization of the fraction of the FDp reference covered and the average depth of all dataset points was performed in R (version 2022.12.0+353) using the *ggplot2* package.

#### Evaluation and comparison of *de novo* assemblies

*De novo* assemblies of reads from datasets obtained using different protocols were performed to assess the efficiency in *de novo* reconstruction of the phytoplasma genome. For this comparison, one replicate of each protocol (A–F) with the largest normalized read dataset size (4.8 billion nucleotides) was used. The Illumina sequencing datasets (protocols A–E) were assembled using SPAdes version 3.14.1 [56] with default parameters. For the nanopore sequencing dataset (protocol F), assemblers designed for long reads were used. Initially, two *de novo* assemblers were compared on the complete dataset from protocol F: Flye [57], which was previously used for the *de novo* assembly of the FDp genome [17], and the *De Novo* Assemble Long Reads tool in CLC Genomics Workbench (Qiagen, Hilden, Germany), with parameters detailed in Material S1. As the CLC assembler produced superior results (Material S2), it was used for further analyses of dataset F. The assemblies were evaluated using the genome Quality Assessment Tool (QUAST, version 5.2) [58] with the FDp reference genome (CP097583). QUAST was used to generate comparative diagrams of *de novo* assemblies from different protocols.

We further investigated the performance of *de novo* assembly of phytoplasma genomes using datasets generated with the protocol that yielded the highest fraction of phytoplasma reads and included Illumina sequencing (D) and a protocol with nanopore sequencing (F). For these comparisons, genome assemblies were performed on subsampled datasets of different sizes, as well as on a mixed dataset to also test a hybrid assembly mode (assembling Illumina and nanopore sequencing data together). The datasets obtained with protocols D and F were downsized to 2.4, 4.8 and 6 billion nucleotides in 5 replicates. For each replicate, *de novo* assembly was performed as described above for Illumina or nanopore sequencing data. For the hybrid assembly, the SPAdes hybrid option (version 4.0.0., default settings) was used for datasets of the same sizes, with half of the data (1.2, 2.4 and 3 billion nucleotides) coming from the dataset generated with protocol D and the other half from the dataset generated with protocol F (1.2, 2.4 and 3 billion nucleotides). All assemblies performed were compared in QUAST, and the results were displayed in a diagram in R as described above. The quality metrics of the assemblies and contig lengths were plotted in a ridgeplot group distribution in R.

To perform a reference-independent comparison of the *de novo* assembled genomes, the assembled contigs were also analysed using the Basic Local Alignment Search Tool (blast) to identify all contigs belonging to FDp. Contigs generated from a randomly selected repeat of the *de novo* assembly using the largest comparable dataset (6 billion nucleotides) for each of the three approaches (D, F and hybrid) were analysed. First, a blastn search was performed using a custom phytoplasma database created by downloading all nucleotide sequences associated with the search term txid33926[Organism] from the NCBI database on 3 October 2024. All contigs with blast hits were then subjected to a second blastn search against the complete NCBI nt database (on 19 February 2023). The resulting output files were used for taxonomic classification of the contigs using MEGAN (version 6.25.10), and the contigs identified as hits for ‘*Candidatus* Phytoplasma vitis’, 16SrV Elm yellows group and ‘*Ca*. P vitis’ contigs incorrectly assigned to the unclassified NCBI group were further analysed.

The final assembly of the Slovenian FDp isolate was performed using the whole dataset of Illumina reads from protocol D and the whole dataset from nanopore sequencing (protocol F) in hybrid assembly with the SPAdes hybrid mode (version 4.0.0, default settings). Additionally, the Illumina reads obtained with protocol D were remapped to contigs (parameters in Material S1), and corrected consensus sequences of contigs were extracted. This corrected assembly was evaluated against the FDp genome reference (CP097583) using QUAST, as mentioned above, and the average nucleotide identity (ANI) value was calculated using FastANI version 1.34 [59] with the FDp reference genome. The final draft genome was submitted to GenBank (under BioProject PRJNA1231790).

## Results

### Characterization of plant material

The grapevine and hazelnut samples used in this study were confirmed to be infected with FDp using qPCR. The grapevine sample D933/20 used for bioinformatic comparison of the protocols was determined to be genotype M54 [60] based on *map* genotyping with Sanger sequencing of the nested PCR product (GenBank accession number PV085772). The only available FDp reference genome (CP097583), which was also used as a reference in our analyses, corresponds to the same *map* genotype.

### DNA concentrations and qPCR results

The total DNA yield varied considerably depending on the extraction and enrichment methods used in the preparation of sample D933/20 with different protocols (Table 1). The CTAB extraction method (protocol B) yielded significantly more DNA than the KingFisher method (protocol A), while the enrichment steps, including differential centrifugation (protocol C) and post-extraction treatment (protocol D), reduced total DNA yield. The qPCR analysis confirmed the successful extraction of FDp in all protocols. The relationship between the qPCR values of the 16SrV phytoplasma-specific assay and the 18S rRNA host DNA marker illustrated the effects of enrichment: a lower 16SrV Cq value indicated a higher phytoplasma DNA concentration, while a higher 18S rRNA Cq value reflected a depletion of host DNA. This inverse relationship indicates that the enrichment steps effectively increase the proportion of phytoplasma DNA while minimizing the presence of host DNA (Table 1). The additional step of WGA (protocol E and F) increased the overall DNA yield by amplifying both phytoplasma and host DNA (Table 1). Nanodrop measurements of DNA concentration and purity (A260/280 and A260/230) and Qubit DNA concentration values can be found in the Material S3 for the different steps of each protocol. Negative controls confirmed the absence of contamination.

### Evaluation of enrichment consistency using qPCR

To evaluate the consistency of the enrichment protocols and their applicability to different hosts, we tested four additional 16SrV phytoplasma-infected samples – two from grapevine and two from hazelnut – using three protocols selected to represent the methodological range: a rapid extraction (protocol A), a pre-extraction enrichment (protocol C) and a combined pre- and post-extraction enrichment approach (protocol D).

qPCR analysis confirmed that the enrichment approaches shown to be effective in sample D933/20 were also effective in other grapevine samples, as well as in hazelnut samples from both leaf and root tissue (Material S4). The level of host DNA, as determined by the 18S rRNA assay, decreased consistently with each additional enrichment step, as indicated by increased Cq values: between protocols A and C, 18S Cq increased by 2.0–4.1 cycles, and between protocols C and D by an additional 3.5–8.6 cycles (Material S4). Meanwhile, the phytoplasma signal measured by an assay specific to the 16SrV phytoplasma group remained approximately the same, with Cq changes between protocols ranging from −1.6 to 1.3, indicating stable phytoplasma DNA content during enrichment (Material S4).

### Evaluation of different protocols based on mapping to the FDp reference genome using subsampled datasets

The sequencing runs performed with different protocols (A–F) with sample D933/20 yielded outputs in the range of 4.8–7.4 billion nucleotides (Table 1). The quality control with TapeStation of the high-molecular-weight DNA and the final preparation of the library confirmed the suitability for nanopore sequencing (Material S5). This resulted in the highest output achieved with MinION nanopore sequencing, with DNA CS confirming the successful sequencing run (Material S5). Mapping these complete datasets from different protocols to the FDp reference revealed distinct distributions of mapped read lengths. As expected, for the Illumina sequencing runs, the mean read length was between 146.98 and 149.36 bp, according to the sequencing mode, while for the nanopore sequencing, it was 5,818.82 bp (Material S6).

To ensure comparability, all datasets of the different protocols were randomly subsampled to the smallest dataset size and compared based on the percentage of nucleotides mapped to the host genome (*V. vinifera*) and the FDp reference genome (CP097583). Protocols A and B, which did not include any enrichment step, yielded the highest percentage of reads mapping to the host genome (Table 1). Among these protocols, protocol A yielded the lowest percentage of phytoplasma reads. Changing the DNA extraction method from KingFisher (protocol A) to CTAB (protocol B) slightly improved the yield of phytoplasma reads by 1.3-fold (Table 1). The inclusion of an enrichment step prior to CTAB extraction (protocol C) led to a further increase in phytoplasma reads and yielded almost double the relative phytoplasma reads compared to protocol B (Table 1). Protocol D showed the most significant improvement and achieved a 4-fold increase in phytoplasma reads compared to protocol C (Table 1). This enrichment resulted in complete coverage of the FDp reference genome with an average depth of 19× and the longest consensus sequence among all protocols tested at the normalized subsample size. Incorporating WGA in protocol E maintained complete genome coverage and produced a high-quality consensus sequence but resulted in a slightly lower (1.5-fold) percentage of phytoplasma reads compared to protocol D (Table 1), while the percentage was similar (1.1-fold lower) to protocol E when using nanopore sequencing (protocol F). While WGA also increased the percentage of host reads, protocols E and F still achieved 12-fold and 10-fold average depth of the FDp reference genome at the normalized subsample size, respectively.

**Fig. 2.**
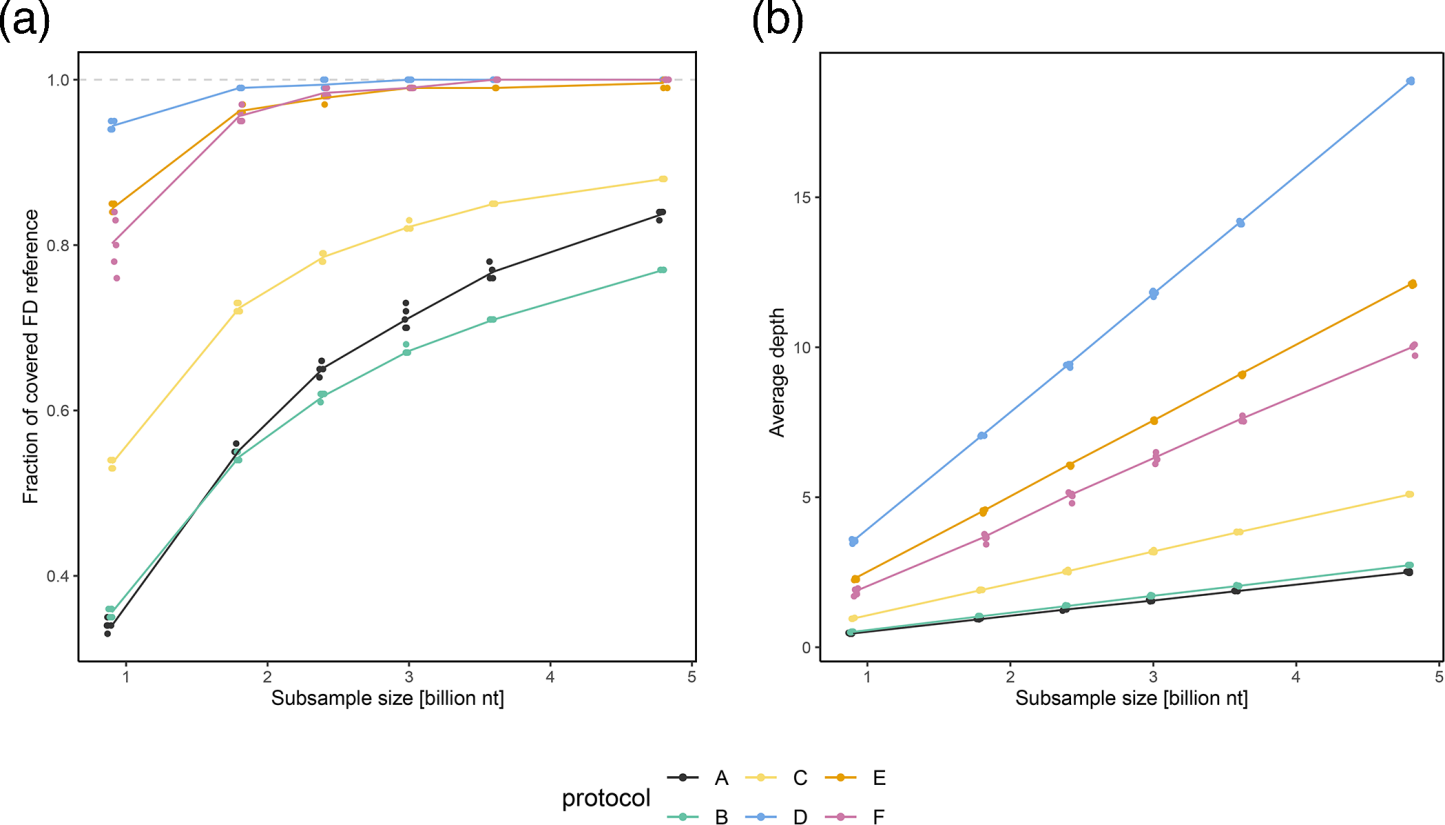
Rarefaction analysis showing mapping results at 6 different subsampled dataset sizes (0.9, 1.8, 2.4, 3, 3.6 and 4.8 billion nucleotides) for 6 compared sample preparation protocols (A–F, colour-coded according to the legend). For each protocol/subsample dataset size combination, analysis was performed in five replicates (each dot represents the result for a single replicate). (**a**) Proportion of the FDp reference genome covered as a function of a subsample size. (**b**) Average depth of the FDp reference genome as a function of a subsample size.

Performing a rarefaction analysis, in which randomly subsampled datasets (at six dataset sizes) were analysed in five replicates, allowed an unbiased comparison of the performance of the different protocols (A–F) when mapping to the FDp reference genome (Fig. 2a). Across all protocols, the proportion of the average depth of the FDp reference genome increased with the size of the sequencing data subsample (0.9–4.8 billion nucleotides), although the rate of increase varied considerably between protocols (Fig. 2b).

Protocols D, E and F performed best and achieved comparable genome coverage of 100% for D and 99% for E and F with a subsample size of 3 billion nucleotides (Fig. 2a). At this subsample size, the sequences of protocol A had an average genome coverage of 71%, protocol B 67% and protocol C 82%. Below this dataset size, protocol D provided better reference genome coverage than E and F (Fig. 2a). Protocols A and B performed the worst and did not achieve the genome coverage achieved by D, E and F, even at the largest subsample size (Fig. 2a). Protocol C, which included only one enrichment step of differential centrifugation, showed intermediate performance. The inclusion of enrichment steps (protocols C–F) contributed to higher genome coverage, with additional enrichment after extraction (D–F) resulting in better performance than enrichment before extraction alone (C) (Fig. 2a). For all protocols, a clear linear increase in average depth was observed with increasing dataset size (Fig. 2b). However, protocol D showed a steeper increase in average depth than the others (Fig. 2b). In contrast, protocols A and B showed a minimal improvement in average depth with larger datasets (Fig. 2b).

### Evaluation of different protocols based on quality of genome assembly of subsampled datasets

To determine the most suitable *de novo* assembler for long-read sequencing data, two assemblers were tested with the full-size nanopore sequencing dataset (protocol F) and evaluated using QUAST (Material S2). The *De novo* Assemble Long Reads tool in CLC Genomics Workbench outperformed Flye and covered a higher fraction of the FDp reference genome with fewer contigs (Material S2). Based on these results, the CLC tool was selected for further comparisons.

**Fig. 3.**
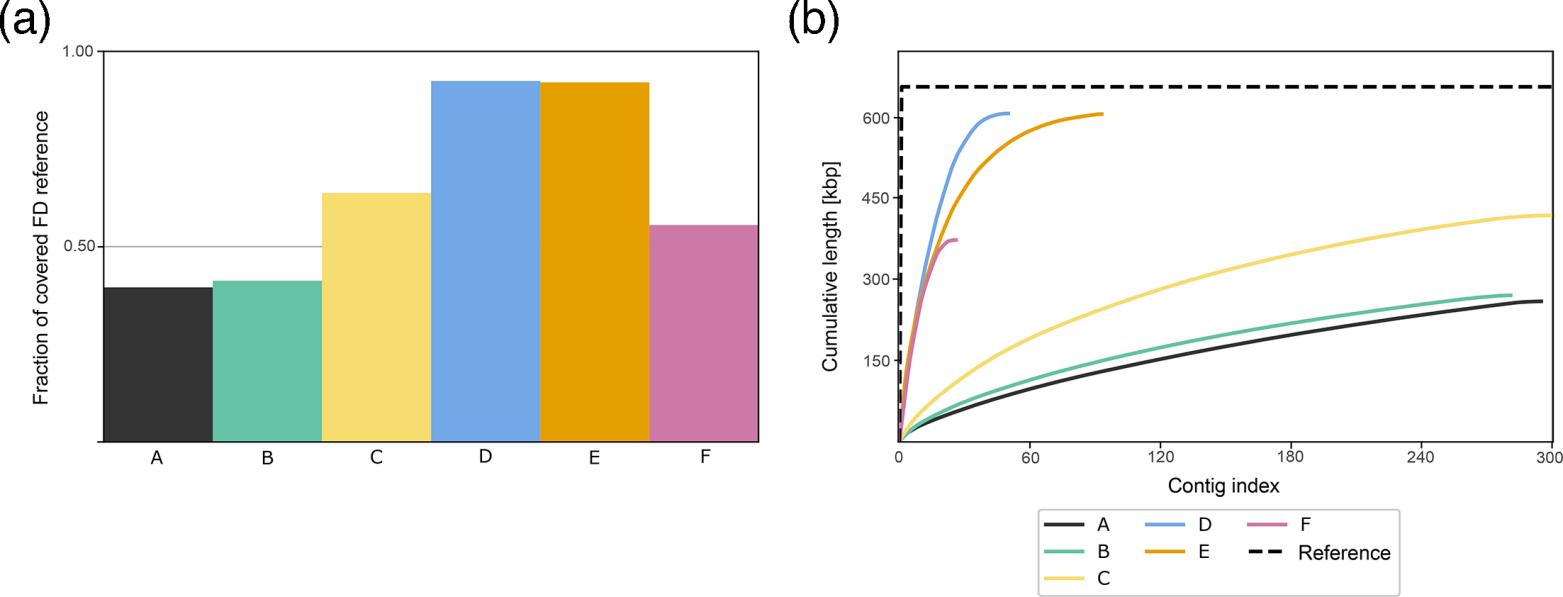
QUAST-based quality metrics for the *de novo* assemblies of randomly subsampled datasets (size of 4.8 billion nucleotides) of different sample preparation protocols (A–F, colour-coded according to the legend on the right, on both panels). (**a**) The proportion of the FDp reference genome covered with assembled contigs. (**b**) Cumulative plot from QUAST showing the total assembled phytoplasma contig length as contigs are added sequentially from longest to shortest (the contig index represents the position of contigs sorted by length, with lower indices corresponding to longer contigs and higher indices to shorter contigs). kbp, 1,000 bp.

Subsequently, randomly subsampled datasets of equal size (4.8 billion nucleotides) from protocols using Illumina sequencing (A–E) were assembled using SPAdes, while the nanopore subsample was assembled with CLC Genomics Workbench, and all assemblies were compared in QUAST (Material S7). The results showed significant differences in reference genome coverage and assembly quality between the protocols (Fig. 3a). Protocols A and B, in which no enrichment step was performed, resulted in assemblies in which less than 50% of the reference genome was covered by the assembled phytoplasma contigs. In contrast, the addition of a simple enrichment step, such as differential centrifugation (protocol C), resulted in assemblies with genome coverage improved to 64%. Protocols D and E with additional enrichment steps after DNA extraction resulted in assemblies that achieved over 90% of the reference genome coverage.

Protocols A–C resulted in more than 270 contigs mapped to the reference genome, whereas protocols D–F yielded fewer than 100 contigs, reflecting a lower degree of fragmentation (Material S7). The steep curves of the cumulative plot of aligned contigs indicate that protocols D, E and F contained long contigs that quickly accumulate a large portion of the reference genome (Fig. 3b). Looking at the NGA50 values (Material S7), i.e. the length of the shortest contig that, together with longer contigs, covers at least 50% of the reference genome, protocol D shows the best performance with longer and more continuous contigs (NGA50=18,116). Protocols E and F have lower NGA50 values (11,880 and 10,569, respectively). Protocol F, which used the same enrichment and WGA sample as protocol E but was sequenced using nanopore sequencing technology, achieved lower genome coverage and assembly quality for this subsampled dataset size. The largest alignment was 12,895 nucleotides shorter than the largest alignment achieved with protocol E (Material S7). In summary, the *de novo* assembly of the FDp genome was most successful using the dataset obtained with protocol D.

### Comparative analysis of assembly performance across best-performing sample preparation protocols including Illumina and nanopore sequencing and their hybrid mode

**Fig. 4.**
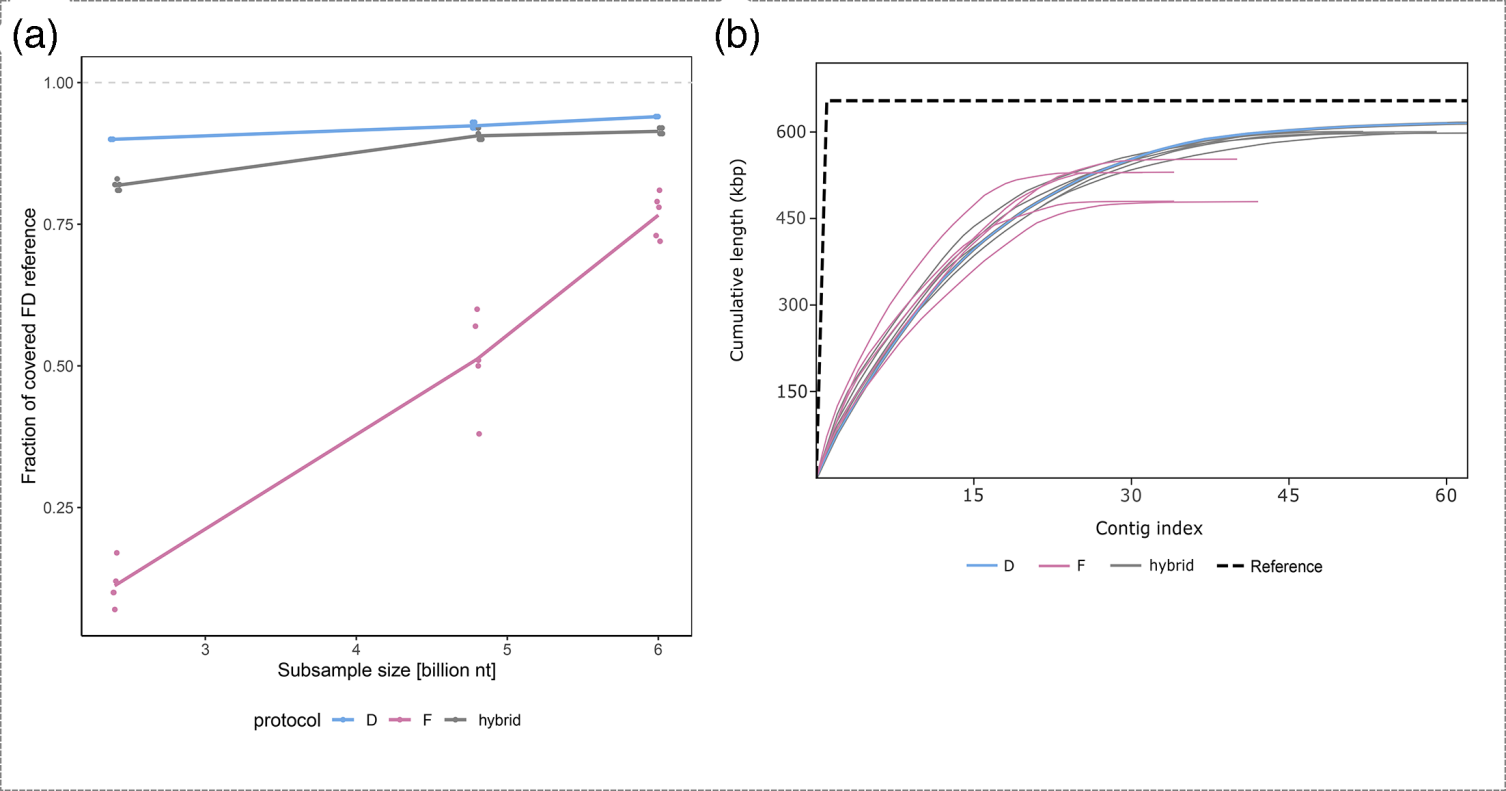
(**a**) Rarefaction analysis showing FDp reference genome coverage by contigs from *de novo* assemblies generated using protocols D and F and their hybrid assembly (combining equal parts of data from protocols D and F). Assemblies are colour-coded according to the legend and analysed at 3 subsampled dataset sizes (2.4, 4.8 and 6 billion nucleotides). Each dot represents the result of a single replicate, with five replicates per assembly and dataset size combination. (**b**) Cumulative plot from QUAST for assemblies of 6-billion-nucleotide datasets using protocols D and F and their hybrid mode (colour-coded according to the legend); the contig index represents the position of phytoplasma contigs sorted by length, with lower indices corresponding to longer contigs and higher indices to shorter contigs.

To further evaluate the *de novo* assemblies, protocol D (the best-performing protocol including Illumina sequencing), protocol F (protocol including nanopore sequencing) and their hybrid mode assembly (D and F) were compared across 3 dataset sizes: 2.4, 4.8 and 6 billion nucleotides (Fig. 4). For the hybrid assemblies, half of the input data were sampled from Illumina and the other half from the nanopore sequencing dataset. Each *de novo* assembly was repeated five times with randomly subsampled datasets. The *de novo* assembly from nanopore sequencing datasets (protocol F) was strongly influenced by random subsampling and dataset size, which affected the coverage of the FDp reference genome by contigs (Material S8). However, this effect was much less pronounced in the hybrid assembly, as shown by the coefficient of variation of genome coverage between replicates for the largest dataset size (0.05 for protocol F compared to 0.004 for the hybrid assembly) (Material S8).

At the smallest dataset size (2.4 billion nucleotides), the phytoplasma contigs obtained from the nanopore sequencing dataset (F) covered only a small portion (7–17%) of the reference genome (Fig. 4). Coverage improved with increasing dataset size but remained lower than the coverage of protocol D and hybrid mode assemblies. In contrast, assemblies from datasets obtained with protocol D consistently achieved the highest genome coverage, exceeding 90% even for the smallest dataset (Fig. 4). Hybrid assemblies performed better than protocol F but did not outperform protocol D. They showed moderate genome coverage at smaller dataset sizes and approached the performance of protocol D at the largest dataset (Fig. 4).

Assemblies of datasets using protocol D produced fewer phytoplasma contigs than the hybrid protocol at a subsample size of 2.4 billion nucleotides (an average of 97 with protocol D and 227 with the hybrid mode), but the opposite happened at the largest subsample size (protocol D had an average of 60 contigs and the hybrid mode 46) (Material S8). Protocol F consistently produced the lowest number of phytoplasma contigs, averaging 24 with the 6-billion-nucleotide big dataset (Material S8). The cumulative plot of the QUAST analysis of the 6-billion-nucleotide datasets showed that the assemblies from nanopore and hybrid approaches produced longer phytoplasma contigs in some replicates than those obtained with protocol D (Fig. 4b). Nevertheless, at the 6-billion-nucleotide dataset point, the assemblies using protocol D produced an average of 60 contigs covering 94% of the reference genome, while the hybrid assemblies averaged 46 contigs covering 92% (Material S8).

**Fig. 5.**
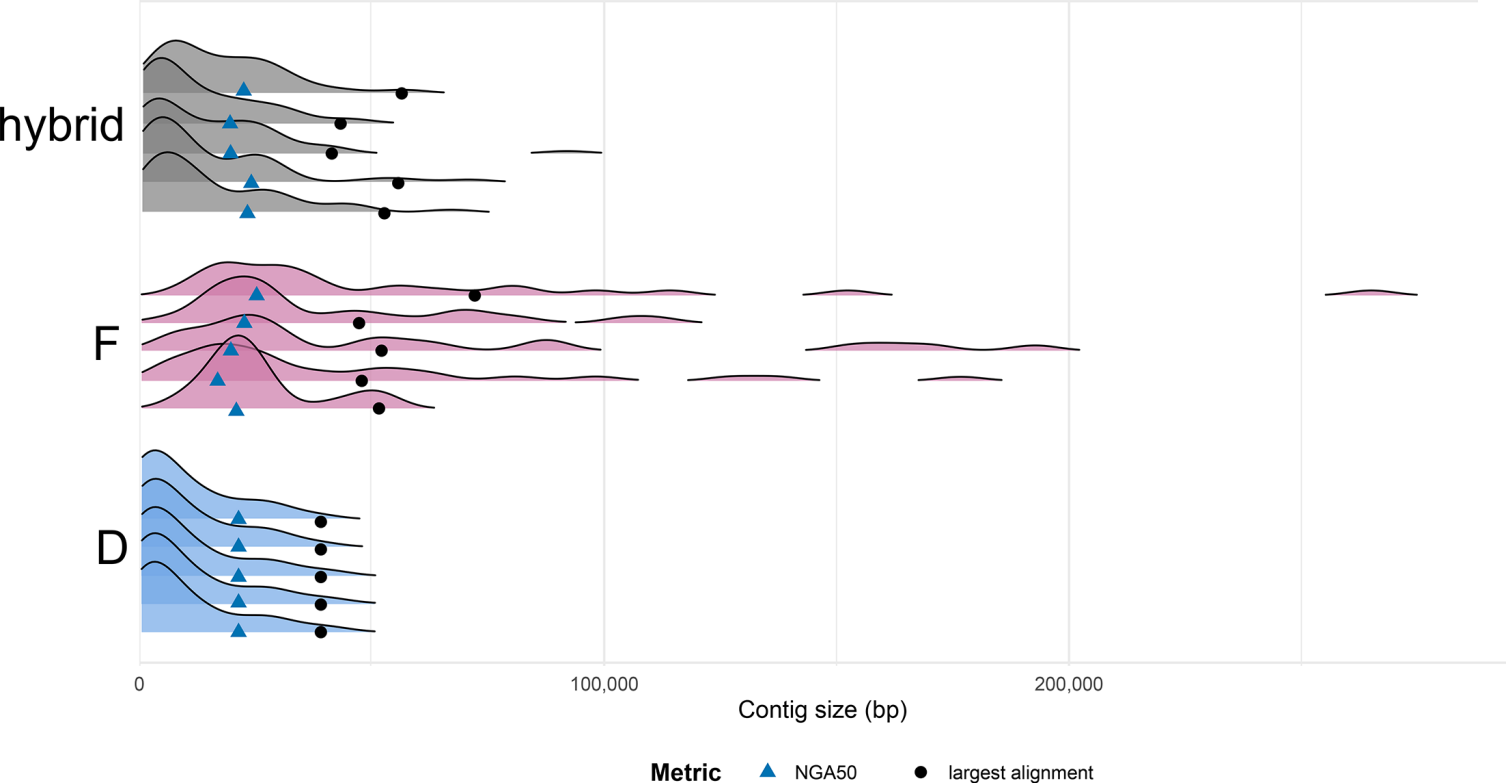
Contig length distributions and assembly quality metrics (calculated by QUAST) for 6-billion-nucleotide datasets assembled using protocol D, protocol F and their hybrid mode in five replicates each. Contig length distributions are shown as ridgeline density plots, where the x-axis represents contig size and the y-axis indicates the estimated probability density within each assembly repetition. The height of each curve reflects the relative probability density, not absolute counts, and is scaled independently for each group to allow visual comparison of distribution shapes. Assembly quality metrics include NGA50 (blue triangles) and the largest alignment for each assembly (black dots).

To assess whether nanopore sequencing read assembly or hybrid assembly results in longer phytoplasma contigs, we compared the distribution of phytoplasma contig lengths in different datasets (D, F and hybrid) for a 6-billion-nucleotide subsample size (Fig. 5 and Material S9). The protocol D assemblies yielded relatively uniform phytoplasma contig lengths across replicates, with ~60% of the contigs in each replicate exceeding ~4,000 bp. In contrast, protocol F assemblies yielded fewer but generally longer contigs, all exceeding 5,000 bp, although these assemblies exhibited high variability between replicates, particularly in maximum contig length (Fig. 5). The hybrid assemblies contained features of both approaches and had a wide range of contig sizes, from ~500 bp to ~91,600 bp (Fig. 5 and Material S9).

Metrics for assembly quality varied between the three sequencing approaches when considering assemblies from datasets containing 6 billion nucleotides. Protocol D resulted in consistent NGA50 values between repeats and the shortest longest alignment, with low misassembly and mismatch rates (Fig. 5 and Material S8). Protocol F produced fewer phytoplasma contigs, had greater variability in NGA50 values and longest alignment lengths between 72,154 and 47,265 but had the highest mismatch and misassembly rates (Fig. 5 and Material S8). Hybrid assemblies had intermediate NGA50 values and longest alignment lengths between 56,110 and 41,063, with mismatch and misassembly rates higher than protocol D but lower than protocol F (Fig. 5 and Material S8).

To determine whether a reference-free analysis of the assembled phytoplasma contigs could reveal additional genomic regions that differ from the reference, an additional blast analysis was performed to search for possible additional FDp contigs within the assemblies. This analysis focussed on a random replicate of each assembly of the largest datasets obtained with the three protocols (D, F and hybrid). When assembling the datasets obtained with protocol D and the hybrid assembly, blast identified more contigs than reported by QUAST (140 contigs in protocol D compared to 61 found by QUAST, and 42 contigs in the hybrid assembly compared to 37 found by QUAST). However, all but one of the additional contigs identified by blast were short (<500 bp) and were therefore excluded by QUAST, which only considers contigs ≥500 bp. An additional contig identified by blast in the hybrid assembly was a hybrid of sequences from *V. vinifera* (26% query coverage, 95.72% identity) and FDp (40% query coverage, 95.42% identity). When assembling the dataset obtained with protocol F, blast did not identify any additional contigs beyond those reported in the QUAST analysis.

### Slovene isolate of FDp genome

The draft genomic sequence of the FDp isolate (D933/20) analysed in this study was generated using a hybrid *de novo* assembly approach combining complete datasets from Illumina and nanopore sequencing and additionally corrected with Illumina reads mapped to contigs. The addition of the complete nanopore sequencing dataset (dataset size: 7.4 billion nucleotides) to the Illumina-derived data from protocol D (dataset size: 6 billion nucleotides) improved the quality of the genome assembly (Material S10). The 17 assembled contigs from the hybrid assembly covered 96% of the FDp reference genome. The largest aligned contig covered one quarter of the entire genome. The assembly showed good contiguity with a high NGA50, minimal misassembly rates, only three misassembled contigs and no local or scaffold gap misassemblies detected (Material S10). Low mismatch and indel rates are further evidence of the accuracy of the assembly (Material S10). The calculated ANI value of 99.97% shows that the Slovenian isolate is highly similar to the only FDp reference available in GenBank, which was obtained from a grapevine sampled in Switzerland.

## Discussion

Enrichment of phytoplasma DNA from naturally infected host plants would provide a significant improvement for whole-genome sequencing, as phytoplasmas cannot be cultured on media. This study focussed on a grapevine sample infected with the FDp M54 *map* genotype, which is efficiently transmitted by *S. titanus* and is the main culprit for epidemics in Slovenian vineyards [51]. The aim of this study was to establish an optimal, rapid and efficient protocol for sequencing the FDp genome directly from infected grapevine tissue. It is known that grapevine samples pose a challenge for DNA extraction due to their high content of polyphenols, polysaccharides and other secondary metabolites. These can co-purify with the DNA and interfere with spectrophotometric purity measurements [61,62], which probably explains the suboptimal A260/280 and A260/230 ratios observed in our study.

Comparison of two protocols without phytoplasma enrichment (A and B) resulted in a similar percentage of mapped reads to the FDp reference genome and had similar performance in the *de novo* assembly of the FDp genome. From a practical point of view, the KingFisher extraction included in protocol A is faster and can be easily scaled up in a semi-automated way [49]. It has been shown to be efficient in processing of grapevine samples and comparable to the CTAB extraction method in downstream analyses such as PCR and real-time PCR [55]. However, due to the lower total DNA yield obtained with the KingFisher extraction, we used the CTAB method for downstream enrichment protocols and additional comparisons (C–F). We did not test how the enrichment steps would affect the DNA extracted with KingFisher. In future applications, incorporating an RNase treatment during the DNA extraction phase could help improve DNA purity and increase quality scores during library preparation. In our case, DNA quantification for comparisons and later downstream applications was based on the fluorometric Qubit measurements, which specifically measure dsDNA and are not affected by the presence of RNA.

Differential centrifugation is a simple and effective method for the enrichment of phytoplasma DNA, addressing the challenge of low phytoplasma concentrations in grapevine samples. As previously reported by Palmano [63], this method yields higher amounts of phytoplasma DNA compared to CTAB extraction alone – a finding initially based on PCR analyses and which we confirmed using HTS, allowing the analysis of larger genome segments. Differential centrifugation (protocol C) enhanced genome assembly by increasing the average coverage of reads mapping to the FDp reference genome and improving genome coverage compared to CTAB extraction alone (protocol B). This simple enrichment step, which does not require specialized equipment, also improved *de novo* assembly, enabling contigs to cover a substantial portion of the FD genome even at moderate dataset sizes.

By adding the post-extraction enrichment step, genome coverage and average depth were further improved. In this step, we employed the NebNext Microbiome Enrichment Kit to separate prokaryotic and eukaryotic DNA using the methyl-CpG binding domain protein [64]. This method selectively depletes plant host DNA, which naturally undergoes CpG methylation, while the non-methylated DNA of phytoplasmas is retained. The approach has been successfully applied to previous phytoplasma genome assemblies [17,25, 65, 66] and has proven to be a valuable tool for obtaining genomes from natural hosts as well. We included it in protocol D, which demonstrated the highest efficiency in capturing the FDp genome with reads mapping, achieving over 90% coverage even at the smallest dataset size. *De novo* assembly using datasets derived from this protocol outperformed all other protocols, producing the most complete and contiguous reconstruction of the reference genome. Protocol D yielded the highest assembly quality, with minimal errors and the greatest contiguity, highlighting the robustness of short-read Illumina sequencing for high-accuracy assemblies.

To determine whether the two enrichment steps perform consistently across samples, we included additional samples of grapevine and hazelnut infected with 16SrV phytoplasma. Due to resource constraints, these samples were not sequenced; however, we evaluated enrichment efficiency using qPCR assays targeting host DNA (18S rRNA) and phytoplasma DNA (16SrV-specific assay). Both enrichment steps proved to be effective compared to the simplest protocol (protocol A) and resulted in a consistent reduction of host DNA in all samples. Importantly, the enrichment strategy was successful not only in grapevine, but also in hazelnut and even in another tissue matrix (root phloem), suggesting that the method is broadly applicable across hosts and sample types. These results support the robustness and transferability of the enrichment protocol.

To complete the genomes of isolated phytoplasmas, long reads from nanopore sequencing can be used. However, the amount of DNA obtained after using the NebNext Microbiome Enrichment Kit was too low to successfully prepare a library for nanopore sequencing. To solve this problem, we used WGA, which employs multiple displacement amplification. The isothermal amplification reaction has been reported to produce DNA with a low bias in sequence representation and achieve greater genome coverage compared to PCR-based WGA methods [58]. The sample enriched with differential centrifugation, isolated with the CTAB method, further enriched with the NebNext Microbiome Enrichment Kit and then amplified with the REPLIg-midi WGA kit was sequenced using both platforms – Illumina and ONT. The WGA did not significantly alter the end result of the FDp reference genome coverage and enabled the acquisition of high-quality genomic data from limited amounts of DNA, while ensuring compatibility with long-read sequencing technologies that require higher input. The sequencing approach that followed WGA did not affect the percentage of reads that covered the FDp reference genome. Both methods achieved complete coverage of the FDp reference genome with an average depth of more than tenfold, demonstrating their effectiveness for phytoplasma genome analysis.

Nanopore sequencing used in protocol F has demonstrated its strength in generating much longer reads – the longest read that could be mapped to the reference genome was 95,584 bp long. Consequently, assemblers can output longer contigs, with the largest alignments surpassing those from protocol D at higher dataset sizes, highlighting the potential of long-read sequencing for resolving repetitive and structurally complex regions. However, when performing rarefaction analyses for *de novo* assemblies of smaller datasets where genome coverage was minimal and the assemblies contained more mismatches and misassemblies, its limitations became apparent. These results emphasize the need for larger nanopore sequencing datasets to achieve more reliable assemblies, as the lower base-level accuracy of nanopore reads compared to Illumina sequencing remains a challenge.

The hybrid *de novo* assembly approach combines the strengths of Illumina and nanopore sequencing, making it a compelling choice for phytoplasma genome reconstruction. This integration resulted in improved genome coverage and accuracy with fewer mismatches and misassemblies compared to assemblies performed with nanopore reads only (protocol F). In terms of assembly contiguity, the hybrid mode outperformed assemblies performed with Illumina reads only (protocol D) and assembled longer phytoplasma contigs with higher NGA50 at the largest dataset size. However, in hybrid mode, careful post-assembly validation is required to ensure the accuracy of the assembly. This was the case when some chimeric sequences of phytoplasmas and grapevine were detected by an additional blast analysis. To take advantage of both Illumina and nanopore sequencing, we used the hybrid mode to obtain the whole genome of the Slovenian isolate of FDp. Using the entire datasets obtained with protocols D and F in the hybrid assembly of short Illumina and long nanopore sequencing reads enabled the assembly of an almost complete FDp reference genome (96% coverage) with only 17 contigs. Compared to other phytoplasma genome drafts [67], the number of contigs is low, and the N50 value is high, indicating a high quality of assembly. In the future, different assemblers for long and short reads and their hybrid mode could also be benchmarked to further improve the assembly.

Although hybrid assembly offers advantages in terms of the completeness and integrity of the genome, its feasibility depends not only on technological considerations but also on the available financial resources. As sequencing strategies vary greatly in cost, it is important to evaluate the cost-effectiveness of sample preparation and sequencing to determine the most practical approach for a particular study (Material S11). Sample preparation cost can be divided into three main categories: (1) the cost of sample preparation chemicals, (2) the cost of library preparation and sequencing and (3) labour cost. The addition of differential centrifugation increases the costs, but mainly due to an extra hour of labour, not because of notable additional expenses for chemicals. Enrichment with the NebNext Microbiome Enrichment Kit and WGA incurs additional costs and additional time to the overall procedure. For the protocols tested, the total cost without sequencing ranged from just over €100 to nearly €900 per sample, and the hands-on time ranged from 2 to 9 h, depending on the complexity of the steps included (Material S9). However, in our case, the biggest cost differences arose at the sequencing phase. For Illumina sequencing, we outsourced library preparation and sequencing to a commercial provider using a high-throughput system such as NovaSeq. This approach offers a competitive cost of around €100 per sample, especially when sequencing is bundled into large runs, but turnaround times depend on the vendor (we typically obtained Illumina sequencing results within 2 weeks). In our case, in-house nanopore sequencing involved higher upfront costs of around €800 for library preparation and sequencing and required a full day of hands-on work but had the advantage of much faster results (after 3 days). Ultimately, the decision between Illumina and nanopore sequencing depends on factors such as the specific goals of the study, access to in-house equipment, availability of commercial vendors and the timeline of the project. Although the hybrid assembly provides the highest quality, our results show that protocol D alone can provide very high-quality assemblies, making it a sufficient choice considering cost, time and bioinformatics resources.

New approaches to sequencing phytoplasma genomes provide valuable insights. While earlier genome projects using Sanger sequencing faced significant obstacles due to their labour-intensive and time-consuming nature [19], recent advances in HTS technologies have made it possible to generate draft sequences or even complete phytoplasma genomes with greater efficiency [17,24, 29, 34, 35]. Despite the drawbacks of draft genomes, phytoplasma sequence drafts have a significant impact as they expand our understanding by revealing previously undiscovered genetic information in phytoplasma strains and providing new information for the design of new primer sets for use in population genetics and diagnostics. In our study, the best protocol for whole-genome sequencing of FDp included CTAB extraction, pre-extraction enrichment (differential centrifugation), post-extraction enrichment (NEbNext Microbiome Enrichment kit) and Illumina sequencing (protocol D). The data obtained with this protocol were also effectively utilized in hybrid assembly with nanopore sequencing reads and led to a draft genome assembly of the Slovenian FDp isolate. This study shows that the optimized enrichment protocols can facilitate genome sequencing of naturally infected phytoplasma hosts, not only in grapevine but also in other woody plants with similarly low phytoplasma concentrations, as shown in hazelnut samples infected with 16SrV phytoplasma. To date, research of FDp epidemiology has been based on Sanger sequencing of specific genes, especially the *map* gene [51,60, 68,70]. However, our protocol has great potential to enable whole-genome sequencing of a larger number of different isolates of quarantine FDp, which will contribute to a better understanding of the epidemiology and consequently to a more effective implementation of measures to prevent the spread of this dangerous disease.

## Supplementary material

10.1099/mgen.0.001514 Uncited Supplementary Material 1.

## Abbreviations

FD: Flavescence dorée
FDp: Flavescence dorée phytoplasma
ONT: Oxford Nanopore Technologies
WGA: whole-genome amplification
blast: Basic Local Alignment Search Tool
QUAST: Quality Assessment Tool
