# Supplementary material for: From vineyard to genome: optimized enrichment and sequencing of Flavescence dorée phytoplasma from grapevine samples
Source: Microb Genom. 2025 Sep 29;11(9):001514. doi: 10.1099/mgen.0.001514 (PMC12479172; doi:10.1099/mgen.0.001514)
Supplement: Uncited Supplementary Material 1. [file mgen-11-01514-s001.pdf]

Supplementary material

From Vineyard to Genome: Optimized Enrichment and Sequencing of Flavescence dorée Phytoplasma from Grapevine Samples

Zala Kogej Zwitter[1,2], Denis Kutnjak[1], Nataša Mehle[1,3]

[1]National Institute of Biology, Department of Biotechnology and Systems Biology, Ljubljana, Slovenia
[2]Jožef Stefan International Postgraduate School, Ljubljana, Slovenia
[3]School for Viticulture and Enology, University of Nova Gorica, Nova Gorica, Slovenia

Corresponding author: natasa.mehle@nib.si, zala.kogej.zwitter@nib.si

Supplement 1

Table 1: Technical specifications of the servers used for bioinformatic analyses

Parameter	Server 1 – Lenovo ThinkSystem SR665 (Rack Mount)	Server 2 – HP Z6 G4 Workstation
CPU	AMD EPYC 7452 (32 cores, 64 threads) @ 2.35 GHz	Intel Xeon Silver 4216 (16 cores, 32 threads) @ 3.2 GHz
GPU	NVIDIA Quadro RTX 6000/8000	NVIDIA Quadro P2000
RAM	1.08 TB	270 GB
Storage	89.64 TB (ext4)	11.76 TB across multiple partitions (ext4)
Operating system	CentOS Stream 8	Debian GNU/Linux 12 (bookworm)
Kernel version	Linux 4.18.0-490.el8.x86_64	Linux 6.1.0-23-amd64

Table 2: Parameters used for read mapping in CLC Genomic Workbench for Illumina reads

	Mapping to grapevine (GCF_000003745.3)	Mapping to FD reference genome (CP097583)
Match score	1	1
Mismatch cost	2	2
Insertion cost	3	3
Deletion cost	3	3
Length fraction	0.5	0.8
Similarity fraction	0.8	0.95
Auto-detect paired distances	yes	yes
Non-specific match handling	map randomly	map randomly

Table 3: Parameters used for read mapping in CLC Genomic Workbench for nanopore reads

	Mapping to grapevine (GCF_000003745.3)	Mapping to FD reference genome (CP097583)
Enable long-read spliced alignment	no	no
Match cost	1	1
Mismatch cost	6	6
Gap open cost	4	4
Gap extended cost	2	2
Long gap open cost	24	24
Long gap extended cost	1	1
Score bonus for global alignment	0	0

Table 4: parameters for De Novo Assemble Long Reads in CLC genomic workbench

Contig polishing	Yes, polish with reads
Minimum contig length	1,000
Keep circular contings	yes

Table 5: parameters used for mapping reads to contigs in CLC Genomic Workbench

Match score	1
Mismatch cost	2
Linear gap cost	yes
Insertion cost	3
Deletion cost	3
Length fraction	0.8
Similarity fraction	0.95
Map randomly	yes

Command lines used in the article:

Guppy

```
./guppy_basecaller -i /files/fast5 -s /files/fastq --flowcell FLO-MIN106 --
kit SQK-LSK110 --device "cuda:0" --chunk_size 1000 --chunks_per_runner 256
--gpu_runners_per_device 64
```

NanoFilt

```
NanoFilt nanopore_merged.fastq --headcrop 50 --tailcrop 50 -l 50 | gzip >
nanopore_merged_nanofilt.fastq.gz
```

SPAdes

```
spades.py --12 input.fastq -o file_name
```

Flye

```
flye --nano-raw merged_nanofilt.fastq --out-dir /Flye
```

Quast

```
quast.py contigs.fasta -r CP097583.fasta
```

SPAdes hybrid

```
spades.py --12 Illumina_input.fastq  --nanopore nanopore_input.fastq -o
/SPAdes_hybrid
```

blastn

```
blastn -query contigs.fasta -db /phy_db_oct24 -out blast_to_phy.out  -
outfmt 6
```

fastANI

```
fastANI -q contigs.fasta -r CP097583.fasta -o fastANI
```

Supplement 2

Comparison of QUAST results using the FD genome reference (CP097583) for assemblies of the complete nanopore sequencing dataset generated with Flye and the De Novo Assembly tool for long reads in CLC Genomics Workbench; acronyms and description of the metrics can be found in the QUAST manual (web page).

Genome statistics	CLC_assembly	Flye_assembly
Genome fraction (%)	86.944	61.785
Number of mapped contigs	23	42
Duplication ratio	1.042	1.099
Largest alignment	84674	29200
Total aligned length	591863	443422
NG50	139764	50393
NG75	131461	44311
NGA50	33289	3847
NGA75	23637	-
LG50	3	6
LG75	4	10
LGA50	7	34
LGA75	13	-
**Misassemblies**		
# misassemblies	8	9
# relocations	8	9
# translocations	0	0
# inversions	0	0
# misassembled contigs	6	8
Misassembled contigs length	213902	101547
# local misassemblies	8	1
# scaffold gap ext. mis.	0	0
# scaffold gap loc. mis.	0	0
# unaligned mis. contigs	0	0
Unaligned		
# fully unaligned contigs	1201	6980
Fully unaligned length	28073705	26047895
# partially unaligned contigs	11	0
Partially unaligned length	840337	0
**Mismatches**		
# mismatches	757	188
# indels	1498	562
Indels length	3774	1102
# mismatches per 100 kbp	133.09	46.51
# indels per 100 kbp	263.36	139.04
# indels (<= 5 bp)	1444	550

# indels (> 5 bp)	54	12
# N's	0	300
# N's per 100 kbp	0	1.13
**Statistics without reference**		
# contigs	1224	7082
# contigs (>= 0 bp)	1224	7196
# contigs (>= 1000 bp)	1223	4983
# contigs (>= 5000 bp)	1190	1395
# contigs (>= 10000 bp)	1165	663
# contigs (>= 25000 bp)	355	95
# contigs (>= 50000 bp)	96	6
Largest contig	167776	70753
Total length	29506490	26492176
Total length (>= 0 bp)	29506490	26521045
Total length (>= 1000 bp)	29505631	25010399
Total length (>= 5000 bp)	29395867	16827523
Total length (>= 10000 bp)	29216545	11748879
Total length (>= 25000 bp)	16103813	3160213
Total length (>= 50000 bp)	7405141	340773
N50	27047	8198
N75	17489	3203
L50	304	830
L75	642	2159
GC (%)	38.28	38.88
**Similarity statistics**		
# similar correct contigs	0	0
# similar misassembled blocks	0	0

Supplement 3

Concentration and purity measurements of DNA from different steps of the protocols (see Figure 1). DNA concentration was measured using the Qubit dsDNA HS kit on a Qubit fluorometer, while concentration and purity (A260/280 and A260/230) were assessed using a NanoDrop ND-1000 spectrophotometer. KF – DNA extraction with KingFisher (Thermo Fisher Scientific, USA); dif.c – pre-extraction enrichment by differential centrifugation; NebNext – post-extraction enrichment with the NebNext Microbiome DNA Enrichment Kit (New England Biolabs, Massachusetts, USA); WGA – whole genome amplification.

Protocol	Measurement of DNA at different steps of protocol	Qubit [ng/μl]	Nanodrop [ng/μl]	A260/280	A260/230
A	KF	3.9	34.1	1.56	0.54
B	CTAB	28.0	88.2	1.92	0.51
C	dif.c. + CTAB	2.2	36.9	1.67	0.75
D	dif.c. + CTAB	4.9	54.2	1.75	0.89
E	dif.c. + CTAB	7.4	68.5	1.84	1.01
D	dif.c. + CTAB + NebNext	2.4	47.7	1.95	1.19
E	dif.c. + CTAB + NebNext	2.9	60	1.96	1.23
E / F	dif.c. + CTAB + NebNext + WGA	386.0	/	/	/

Supplement 4

qPCR results of five samples used to evaluate enrichment consistency comparing protocols A, C and D. The table contains the mean Cq values (quantification cycle) of two technical replicates for both the 18S rRNA assay and the specific 16SrV real-time PCR assay as well as the calculated Cq differences between the protocols; * - sampled root phloem and not leaf veins as in other samples.

	Protocol	A	C	D	A	C	D	difference in Cq value between A and C protocol		difference in Cq value between C and D protocol	
Sample ID	plant	18S rRNA assay [Cq]			16SrV specific assay [Cq]			18S rRNA assay [Cq]	16SrV specific assay [Cq]	18S rRNA assay [Cq]	16SrV specific assay [Cq]
D933/20	grapevine	19.1	21.7	26	28.9	28.3	27.3	2.6	-0.6	4.3	-1.0
D572/25	grapevine	18.5	21.1	29.7	31.1	30.4	30.8	2.7	-0.7	8.6	0.4
D1063/20	grapevine	18.3	21.0	24.6	26.8	25.1	26.4	2.7	-1.6	3.6	1.3
SB21-P-1	hazelnut	17.4	19.4	23.1	27.7	28.5	28.7	2.0	0.8	3.7	0.2
D1461/20	hazelnut*	19.1	23.2	26.7	30.8	31.5	30.2	4.1	0.7	3.5	-1.3

Supplement 5

TapeStation results of 10-times diluted whole genome amplified DNA prepared for nanopore sequencing (1) and final library prepared for sequencing (2), compared to the Genomic DNA ScreenTape Kit (Agilent Technologies, Santa Clara, USA) ladder (L).

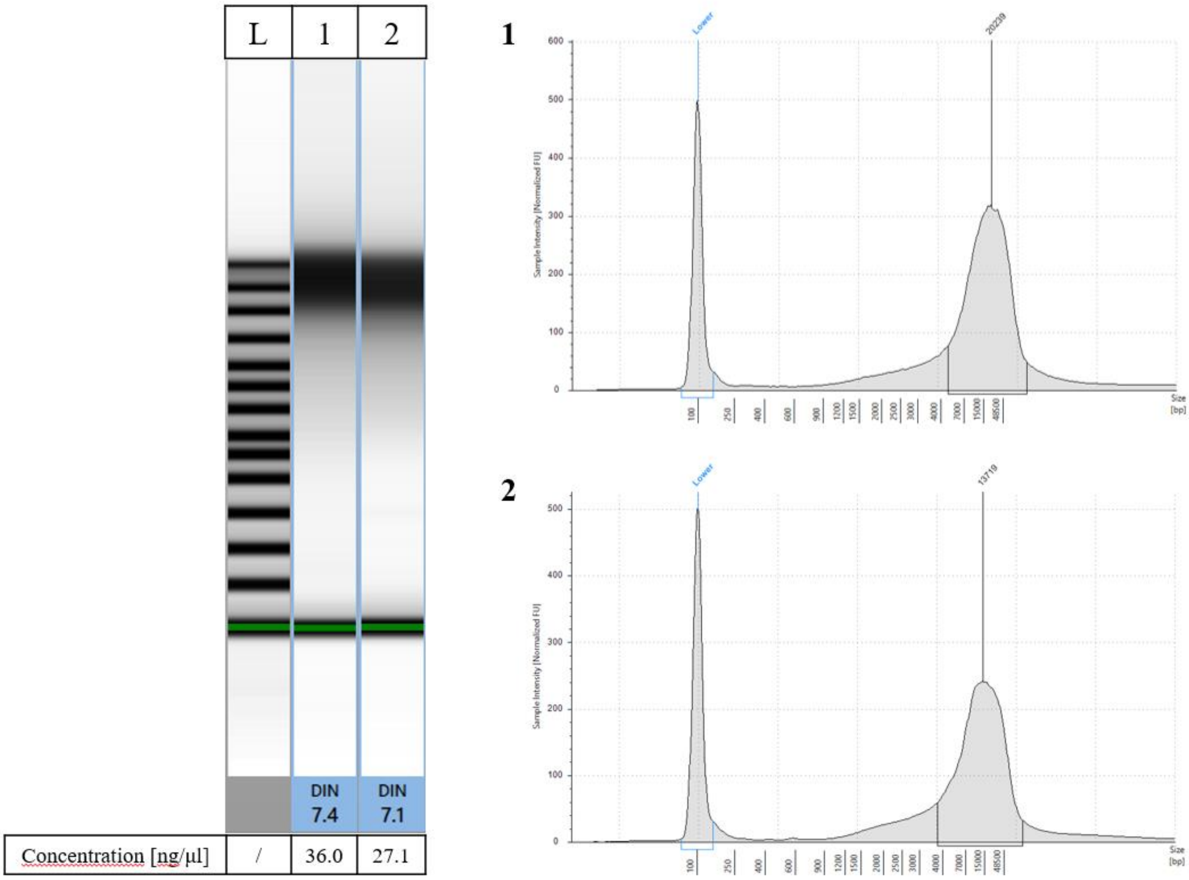

NanoPlot summary statistics report for the nanopore sequencing run performed on a MinION device using an R9.4.1 flow cell for 72 hours, following read trimming uisng NanoFilt (50 bp head and tail crop) and results of mapping to DNA CS (control of sequencing).

General summary:

Mean read length:	3,177.4
Mean read quality:	14.8
Median read length:	1,946.0
Median read quality:	15.0
Number of reads:	2,336,852.0
Read length N50:	5,318.0
Total bases:	7,425,060,521.0

Number, percentage and megabases of reads above quality cutoffs

>Q5: 2336852 (100.0%) 7425.1Mb

>Q7: 2336850 (100.0%) 7425.1Mb

>Q10: 2266229 (97.0%) 7103.0Mb

>Q12: 1951559 (83.5%) 5758.1Mb

>Q15: 1172863 (50.2%) 3039.1Mb

Top 5 highest mean basecall quality scores and their read lengths

1: 90.0 (52)

2: 89.5 (706)

3: 85.2 (109)

4: 30.6 (60)

5: 29.9 (25576)

Top 5 longest reads and their mean basecall quality score

1: 124050 (11.7)

2: 105341 (12.7)

3: 105034 (10.9)

4: 95932 (12.0)

5: 95584 (15.4)

Mapping to DNA CS

Total reference length	3,560
Total read count	26,899
Mean read length	3,254.60
Total read length	87,545,398
Total consensus length	3,558
Fraction of reference covered	1.00
Average coverage	24,693.55

Supplement 6

Summary table of reads statistic mapped to the FD reference genome (CP097583) from whole datasets (protocols A-F) and distribution graphs of mapped read lengths from whole datasets of (1) Illumina sequencing (protocols color-coded in the legend) and (2) nanopore sequencing.

Protocol	Mean read length	Minimum read length	Maximum read length
A	146.98	15	150
B	148.10	15	150
C	148.70	15	150
D	149.36	15	150
E	148.74	15	150
F	5,818.82	59	95,584

1

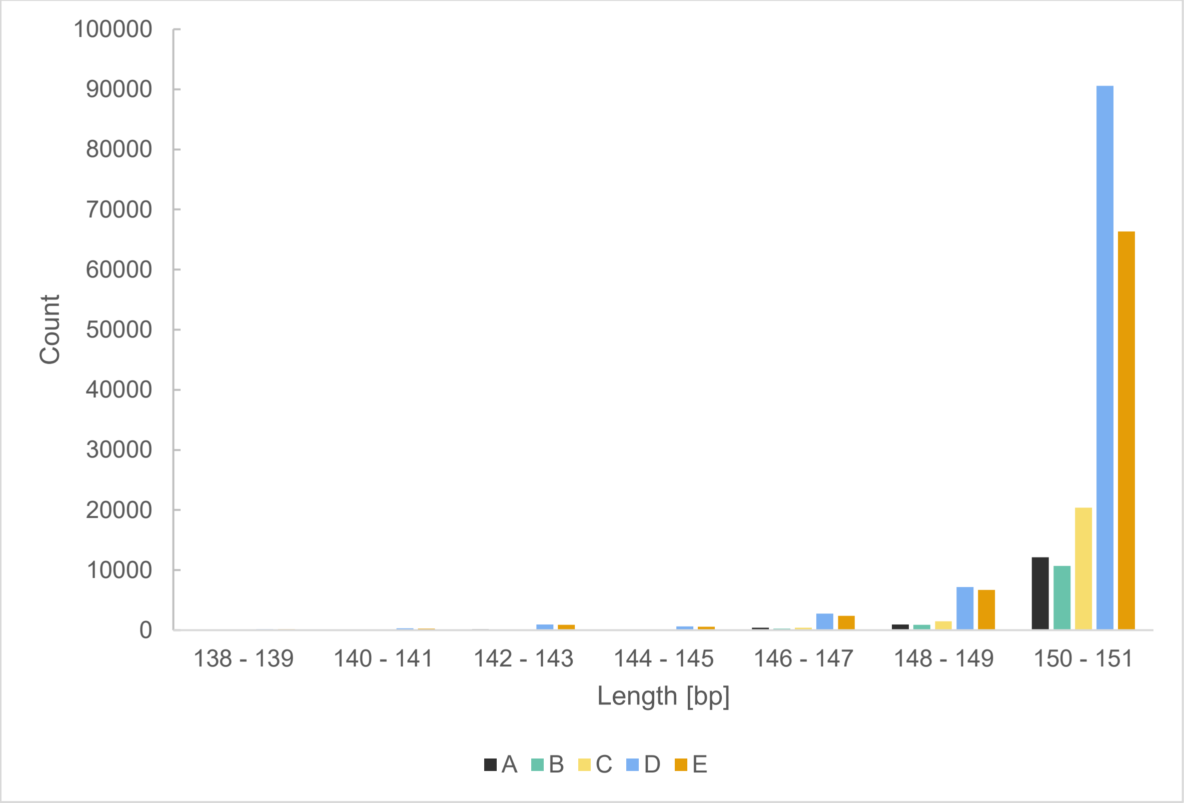

2

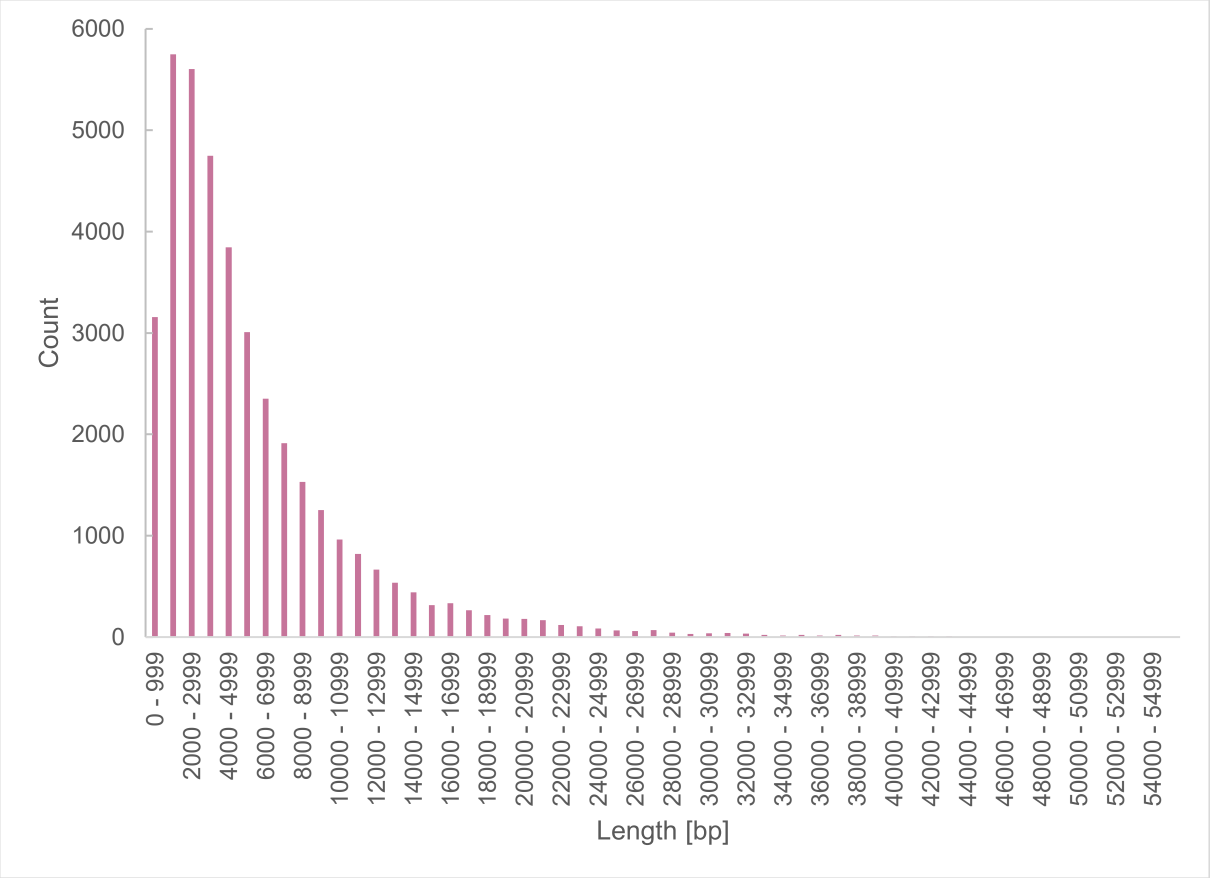

Count
6000
5000
4000
3000
2000
1000
0
0 - 999
2000 - 2999
4000 - 4999
6000 - 6999
8000 - 8999
10000 - 10999
12000 - 12999
14000 - 14999
16000 - 16999
18000 - 18999
20000 - 20999
22000 - 22999
24000 - 24999
26000 - 26999
28000 - 28999
30000 - 30999
32000 - 32999
34000 - 34999
36000 - 36999
38000 - 38999
40000 - 40999
42000 - 42999
44000 - 44999
46000 - 46999
48000 - 48999
50000 - 50999
52000 - 52999
54000 - 54999
Length [bp]

Supplement 7

Comparison of QUAST results using the FD genome reference (CP097583) of *de novo* assemblies of datasets from different protocols (A-F) with dataset sizes of 4.8 billion nucleotides; protocols A-E were sequenced with Illumina and assembled with SPAdes, while protocol F was sequenced with Oxford Nanopore Technology and assembled with the De Novo Assemble Long Reads Tool in CLC Genomics Workbench; acronyms and description of the metrics can be found in the QUAST manual (web page).

Genome statistics	A	B	C	D	E	F
Genome fraction (%)	39.575	41.283	63.753	92.422	92.066	55.526
Number of mapped contigs	286	275	291	43	91	16
Duplication ratio	1	1	1.002	1.001	1.003	1.024
Largest alignment	8066	5379	8388	39045	46472	33577
Total aligned length	259035	270114	417721	605095	603896	371992
NG50	43861	36082	27479	49244	38701	108988
NG90	37979	33699	24070	45314	32054	80007
auNG	3740234	2931348	1759486	1516006	505719	749739
NA50	-	-	-	-	-	-
NA90	-	-	-	-	-	-
auNA	1	1.3	3.4	55.3	125.1	559.9
NGA50	-	-	996	18116	11880	10569
NGA90	-	-	-	2991	1179	-
auNGA	512.1	585.6	1470	19351	16612	12576
LG50	7	9	11	7	8	3
LG90	13	16	21	12	16	6
LA50	-	-	-	-	-	-
LA90	-	-	-	-	-	-
LGA50	-	-	161	13	15	16
LGA90	-	-	-	38	71	-
Misassemblies						
# misassemblies	9	5	10	7	2	9
# relocations	9	5	10	7	1	9
# translocations	0	0	0	0	0	0
# inversions	0	0	0	0	1	0
# misassembled contigs	9	5	8	6	2	4
Misassembled contigs length	12968	5293	22746	230763	24681	137989
# local misassemblies	1	1	0	4	1	0
# scaffold gap ext. mis.	0	0	0	0	0	0
# scaffold gap loc. mis.	0	0	0	0	0	0
# unaligned mis. contigs	0	1	0	0	0	1
Unaligned						
# fully unaligned contigs	98018	107145	137434	150843	68026	635
Fully unaligned length	3,19E+08	3E+08	2,84E+08	2,28E+08	8,63E+07	1,42E+07
# partially unaligned contigs	3	19	60	3	3	6
Partially unaligned length	6714	42291	153122	19367	2260	70212
Mismatches						

# mismatches per 100 kbp	185.69	154.01	126.64	42.47	25	237.91
# mismatches	481	416	529	257	151	885
# indels per 100 kbp	25.48	21.1	20.11	10.41	5.46	405.39
# indels	66	57	84	63	33	1508
# indels (<= 5 bp)	55	49	71	49	26	1476
# indels (> 5 bp)	11	8	13	14	7	32
Indels length	391	310	604	849	227	2498
# N's per 100 kbp	0	0	0	0	0	0
# N's	0	0	0	0	0	0
Statistics without reference	**A**	**B**	**C**	**D**	**E**	**F**
# contigs	98304	107420	137725	150886	68117	651
# contigs (>= 0 bp)	202703	202265	298143	442899	363030	651
# contigs (>= 1000 bp)	71024	74292	87130	65674	22637	651
# contigs (>= 5000 bp)	19113	16757	11089	6249	2070	636
# contigs (>= 10000 bp)	6089	4299	1357	1613	559	618
# contigs (>= 25000 bp)	230	119	17	144	36	187
# contigs (>= 50000 bp)	2	0	0	5	0	44
Largest contig	73923	46256	36578	63895	49920	131084
Total length	3,20E+08	3,01E+08	2,84E+08	2,29E+08	8,69E+07	1,47E+07
Total length (>= 0 bp)	3,50E+08	3,29E+08	3,34E+08	3,21E+08	1,52E+08	1,47E+07
Total length (>= 1000 bp)	3,00E+08	2,77E+08	2,49E+08	1,70E+08	5,62E+07	1,47E+07
Total length (>= 5000 bp)	1,80E+08	1,46E+08	8,20E+07	5,63E+07	1,89E+07	1,46E+07
Total length (>= 10000 bp)	8,88E+07	6,03E+07	1,72E+07	2,51E+07	8,59E+06	1,45E+07
Total length (>= 25000 bp)	6,95E+06	3,48E+06	5,08E+05	4,68E+06	1,19E+06	7,70E+06
Total length (>= 50000 bp)	1,38E+05	0	0	2,77E+05	0	2,92E+06
N50	5889	4831	3085	2047	1506	25799
N90	1313	1134	891	647	591	12326
auN	7658.3	6377.9	4049.2	4329.7	3806.9	33383
L50	15409	17598	26586	25503	12090	174
L90	60136	68246	94873	110400	52078	503
GC (%)	34.14	35.02	35.98	37.1	40.31	37
Similarity statistics						
# similar correct contigs	0	0	0	0	0	0
# similar misassembled blocks	0	0	0	0	0	0

Supplement 8

Comparison of QUAST results using the FD genome reference (CP097583) for *de novo* assemblies of five replicates of protocol D, F and their hybrid datasets of different sizes; protocol D was sequenced with Illumina and assembled with SPAdes, protocol F was sequenced with Oxford Nanopore Technology and assembled with the De Novo Assemble Long Reads Tool in CLC Genomics Workbench and their hybrid datasets (half from Illumina and half from nanopore) were assembled with hybrid SPAdes; nt – nucleotides, CV – coefficient of variation, NGA50 - the length of the shortest contig that together with longer contigs covers at least 50% of the reference genome, kbp - 1,000 base pairs.

Dataset type	Size [billon nt]	Repeat	Contig number	Aligned length	Percentage of covered reference	CV of covered reference	NGA50	Largest alignment	Misassemblies	Mismatches per 100 kbp
D	2.4	1	103	590,825	90.30	0.00	7,608	37,855	10	41.98
D	2.4	2	104	589,867	90.06		7,883	38,769	11	60.59
D	2.4	3	96	590,296	90.07		8,931	27,182	8	43.95
D	2.4	4	83	591,699	90.43		9,405	29,878	13	66.09
D	2.4	5	100	588,485	89.81		8,756	36,988	9	40.00
D	4.8	1	45	605,347	92.48	0.00	18,116	39,045	6	47.77
D	4.8	2	41	605,504	92.58		20,047	67,704	8	51.51
D	4.8	3	44	602,006	92.21		21,775	51,648	5	48.57
D	4.8	4	49	602,676	91.98		17,407	37,855	3	38.39
D	4.8	5	49	606,362	92.66		17,060	51,655	5	57.41
D	6	1	60	615,387	93.93	0.00	21,342	39,047	2	20.34
D	6	2	60	615,387	93.92		21,342	39,047	2	20.34
D	6	3	59	615,096	93.87		21,342	39,047	2	19.21
D	6	4	60	615,652	93.87		21,342	39,044	0	24.10
D	6	5	61	615,360	93.91		21,342	39,044	0	18.39
F	2.4	1	8	46,948	7.02	0.03	-	13,654	5	1278.56
F	2.4	2	10	64,368	9.76		-	11,681	2	627.75
F	2.4	3	13	122,111	17.02		-	15,067	9	852.28
F	2.4	4	10	63,342	9.65			14,729	3	674.86

F	2.4	5	9	84,043	12.48		-	13,492	8	712.80
F	4.8	1	18	400,263	59.76	0.15	11,606	33,575	10	395.92
F	4.8	2	26	334,759	49.76		5,807	30,580	7	273.71
F	4.8	3	27	379,715	57.22		8,841	35,187	15	508.05
F	4.8	4	25	339,212	50.77		4,420	33,510	12	453.98
F	4.8	5	22	259,839	37.94		-	27,856	14	532.61
F	6	1	21	530,267	79.37	0.05	20,865	51,557	6	166.39
F	6	2	30	480,199	71.66		16,838	47,840	12	199.65
F	6	3	23	482,802	72.87		19,683	52,100	11	249.84
F	6	4	27	552,937	81.28		22,581	47,265	12	317.64
F	6	5	24	529,664	77.52		25,237	72,154	7	190.86
hybrid	2.4	1	241	543,511	82.80	0.01	3,517	14,467	15	143.62
hybrid	2.4	2	211	539,334	82.43		3,341	12,981	17	173.38
hybrid	2.4	3	229	534,261	81.46		2,714	14,513	20	202.84
hybrid	2.4	4	218	538,666	81.76		3,040	13,381	15	197.98
hybrid	2.4	5	237	534,801	81.25		2,643	15,012	16	199.61
hybrid	4.8	1	66	593,976	90.37	0.01	14,686	30,921	12	120.94
hybrid	4.8	2	75	592,041	90.47		11,773	34,568	6	79.07
hybrid	4.8	3	72	589,019	90.19		12,870	38,553	6	106.09
hybrid	4.8	4	74	592,667	90.51		14,221	54,829	9	70.09
hybrid	4.8	5	69	601,114	91.72		13,025	42,614	8	97.99
hybrid	6	1	43	602,894	92.07	0.004	23,260	52,712	9	88.49
hybrid	6	2	48	598,196	91.47		24,084	55,696	7	48.96
hybrid	6	3	51	600,373	91.73		19,289	41,063	7	74.98
hybrid	6	4	53	598,528	91.08		19,185	42,953	9	90.79
hybrid	6	5	37	600,621	91.49		22,120	56,110	8	93.56

Supplement 9

Contig size distributions from *de novo* assemblies of 6-billion-nucleotide datasets using protocol D, protocol F, and their hybrid mode (half data from D and half from F), each performed in five replicates.

Assembly protocol	D					F					hybrid				
Repetition	1	2	3	4	5	1	2	3	4	5	1	2	3	4	5
Contig sizes	42750	42750	42778	39044	39044	51578	176703	192712	111630	265000	66686	70700	91671	45954	56498
	39047	39047	39047	37671	37671	51541	137160	170285	104260	152394	46369	56539	41063	43051	42907
	37854	37854	37854	31222	31341	48853	127272	161409	81352	114230	44697	50087	39669	33407	35238
	31222	31222	31341	30799	30799	41751	98032	153314	73770	98176	43066	31011	38276	31823	33004
	30799	30799	30799	28955	28955	27123	80699	88284	68255	82061	35624	27897	30643	30547	29370
	28955	28955	28955	28191	28191	26214	63377	86468	68108	78753	30908	27401	28383	29998	29228
	28266	28266	28266	27090	27090	26022	59349	63598	56177	65901	29406	26759	28198	28556	26912
	27090	27090	27090	25825	25825	23524	53531	57446	47265	57245	28434	26529	26974	26910	26809
	25846	25846	25846	25060	25060	23297	51774	50486	46529	53073	27811	25333	25664	26240	26434
	25060	25060	25060	24607	24607	22517	47840	49496	39343	39632	27075	25011	25179	23242	24576
	23438	23438	23438	23439	23439	21850	39241	31325	28091	36236	24917	24084	24962	21875	23439
	21342	21342	21342	21342	21342	21650	36540	28757	28088	34501	24659	23490	23957	21253	23421
	18210	18210	18210	18248	18248	20872	29703	27325	27793	30978	22193	22267	23397	20610	22120
	17051	17051	17051	18189	18189	19926	27911	24852	27222	30581	21972	21894	22520	19185	21406
	15733	15733	15733	17050	17050	19432	26452	24616	27027	29441	14900	21441	22477	18720	17924
	14165	14165	14165	15568	15568	19294	24616	22997	23461	27237	13886	14293	21402	18147	16911
	13535	13535	13535	14165	14165	17435	24507	19683	22682	23774	12792	13612	19289	15941	16332
	13291	13291	13291	13535	13535	17016	23692	19009	22581	21275	12727	12679	18386	14734	15802
	12693	12693	12693	13291	13291	16700	20737	18735	21399	19274	12693	11952	17221	13068	15704
	10998	10998	10998	12693	12693	10668	17737	11867	20125	17612	12472	10694	16809	12627	15023
	10269	10269	10269	10998	10998	9539	17192	11563	16202	16787	12316	10162	16500	12345	9569
	10117	10117	10117	10233	10269		16838	7457	16199	16658	9961	10118	14186	11113	9552

9860	9860	9860	10194	10233	16677	5479	16152	13790	9293	9652	12734	10777	9336
9588	9588	9588	9860	9860	14735		15946	9586	9020	9048	12559	9739	8826
8012	8012	8012	9525	9525	14024		14547		7584	6986	12537	8813	8427
7367	7367	7525	8012	8012	13347		5820		6467	6625	11894	8265	8250
6708	6708	7367	7525	7367	7114				6271	6056	8935	7451	7719
6671	6671	6708	7367	6708	6690				6212	5763	8080	7400	6814
6670	6670	6671	6708	6671	6402				6141	5477	7208	6361	6762
6519	6519	6670	6671	6670	5485				5554	5446	6467	5925	6052
6119	6119	6519	6670	6519					5183	5273	5455	4704	5261
5022	5022	5022	6519	6119					4421	5197	5326	4570	4744
4681	4681	4681	5022	5022					4419	4455	4544	4438	4471
4447	4447	4447	4447	4681					4077	3468	4291	3828	3725
3830	3830	3830	4110	4447					2991	3424	4085	3762	2382
3810	3810	3810	3830	3830					2642	3209	4049	3678	1869
2609	2609	2404	3810	3810					2487	2992	3763	3340	1639
2404	2404	2348	2404	2404					2318	2877	2766	3293	
2348	2348	2348	2348	2348					2007	2773	2642	3253	
2348	2348	2232	2348	2348					1894	2412	2120	2898	
2232	2232	2056	2132	2132					1829	2047	2046	2896	
2056	2056	1632	1829	1632					1541	1968	1723	2853	
1632	1632	1518	1632	1594					1049	1723	1669	2131	
1594	1594	1334	1594	1518						1522	1648	2031	
1518	1518	1315	1518	1334						1180	1603	1874	
1334	1334	1263	1334	1310						1059	1089	1809	
1315	1315	1056	1310	1267						1049	1049	1729	
1263	1263	1033	1263	1263						1038	880	1723	
1056	1056	765	1146	1146							829	1721	
1033	1033	723	1056	1056							554	1704	
886	886	720	1033	1033								1049	
723	723	654	723	886								981	

720	720	649	720	723	980
649	649	586	649	720	
586	586	553	586	649	
553	553	538	553	586	
538	538	531	538	553	
531	531	514	531	538	
514	514	504	514	531	
504	504		504	514	
				504	

Supplement 10

Comparison of QUAST results using the FD genome reference (CP097583) for *de novo* assemblies of datasets from protocols D, F and their hybrid mode with whole dataset sizes; protocol D was sequenced with Illumina and assembled with SPAdes, while protocol F was sequenced with Oxford Nanopore Technology and assembled with the De Novo Assemble Long Reads Tool in CLC Genomics Workbench and hybrid assembly of whole dataset of both was done with SPAdes hybrid mode; acronyms and description of the metrics can be found in the QUAST manual (web page).

**Genome statistics**	**D**	**F**	**hybrid**
Genome fraction (%)	93.904	76.118	95.89
Number of mapped contigs	61	21	17
Duplication ratio	1.002	1.022	1.003
Largest alignment	39044	77022	160663
Total aligned length	615360	499605	629140
NG50	32518	218093	70806
NG75	31385	218093	31762
NGA50	21342	25796	55713
NGA75	10212	3611	24903
LG50	10	2	3
LG75	15	2	6
LGA50	12	9	3
LGA75	23	20	7
Misassemblies			
# misassemblies	0	8	3
# relocations	0	8	3
# translocations	0	0	0
# inversions	0	0	0
# misassembled contigs	0	5	3
Misassembled contigs length	0	237765	243790
# local misassemblies	1	9	0
# scaffold gap ext. mis.	0	0	0
# scaffold gap loc. mis.	0	0	0
# unaligned mis. contigs	0	0	0
Unaligned			
# fully unaligned contigs	169789	1682	154612
Fully unaligned length	260204875	32405098	280992814
# partially unaligned contigs	0	9	0
Partially unaligned length	0	991143	0
Mismatches			
# mismatches	113	837	144
# indels	36	1153	42
Indels length	379	3142	533
# mismatches per 100 kbp	18.39	168.08	22.95
# indels per 100 kbp	5.86	231.53	6.69

# indels (<= 5 bp)	29	1115	33
# indels (> 5 bp)	7	38	9
# N's	0	0	0
# N's per 100 kbp	0	0	0
Statistics without reference			
# contigs	169850	1703	154629
# contigs (>= 0 bp)	597589	1703	561163
# contigs (>= 1000 bp)	76018	1703	74230
# contigs (>= 5000 bp)	7395	1619	9936
# contigs (>= 10000 bp)	1503	1197	3051
# contigs (>= 25000 bp)	52	360	268
# contigs (>= 50000 bp)	0	95	12
Largest contig	39044	285552	160699
Total length	260820308	33905144	281621987
Total length (>= 0 bp)	370404003	33905144	383267062
Total length (>= 1000 bp)	196319386	33905144	226205937
Total length (>= 5000 bp)	60887118	33577489	96078398
Total length (>= 10000 bp)	21155355	30366687	49169311
Total length (>= 25000 bp)	1540493	17082269	9077511
Total length (>= 50000 bp)	0	8208274	885829
N50	2122	25281	2870
N75	1007	14758	1210
L50	29694	355	21927
L75	75317	797	60590
GC (%)	36.87	38.46	37.62
Similarity statistics			
# similar correct contigs	2	0	2
# similar misassembled blocks	0	0	0

Supplement 11

Detailed information on the cost and time required for each step of all six protocols; the chemicals were purchased in 2021 and 2022 and the prices are true for specific date and specific vendors; the table does not include cost of labour; CLC Genomics Workbench (Qiagen, Hilden, Germany), a licenced programme that incurs additional costs, was used for the bioinformatic analysis steps.

Protocol	Step	chemical	Price per package/ procedure [€]	Number of samples for this price	Price per sample [€]	Total price for one sample processed with specific protocol [€]	Hours of work
A	DNA extraction	chemicals for extraction using KingFisher	797.0	53	15.0	122.5	2h hands on
	sequencing	Illumina library preparation & sequencing	430.0	4	107.5		outsourced*
B	DNA extraction	CTAB chemicals	320.0	200	1.6	109.1	5h hands on
	sequencing	Illumina library preparation & sequencing	430.0	4	107.5		outsourced*
C	pre-extraction enrichment	PGB buffer	8.0	25	0.3	109.4	7h hands on
	DNA extraction	CTAB chemicals	320.0	200	1.6		
	sequencing	Illumina library preparation & sequencing	430.0	4	107.5		outsourced*
D	pre-extraction enrichment	PGB buffer	8.0	25	0.3	146.9	
	DNA extraction	CTAB chemicals	320.0	200	1.6		8.5h hands on
	post-extraction enrichment	NEBNext® Microbiome DNA Enrichment Kit	224.9	6	37.5		
	sequencing	Illumina library preparation & sequencing	430.0	4	107.5		outsourced*
E	pre-extraction enrichment	PGB buffer	8.0	25	0.3	178.8	9h hands on + 16 hours of incubation
	DNA extraction	CTAB chemicals	320.0	200	1.6		
	post-extraction enrichment	NEBNext® Microbiome DNA Enrichment Kit	224.9	6	37.5		

	WGA	REPLI-g Midi Kit	799.0	25	32.0		
	sequencing	Illumina library preparation & sequencing	430.0	4	107.5		outsourced*
F	pre-extraction enrichment	PGB buffer	8.0	25	0.3	878.0	9h hands on + 16 hours of incubation
	DNA extraction	CTAB chemicals	320.0	200	1.6		
	post-extraction enrichment	NEBNext® Microbiome DNA Enrichment Kit	224.9	6	37.5		
	WGA	REPLI-g Midi Kit	799.0	25	32.0		
	ONT sequencing	Genomic DNA Reagents	221.4	105	2.1		
		Genomic DNA ScreenTape	320.2	105	3.0		
		T7 Endonuclease I	89.2	16	5.6		
		ONT flowcell	855.0	1	670.3**		
		ONT Ligation sequencing kit	570.0	6	95.0		6h hands on + 72 h of sequencing
		NEBNext® Quick Ligation Module	173.0	20	8.6		
		NEBNext® FFPE DNA Repair Mix	211.0	24	8.8		
		NEBNext® Ultra™ II End Repair/dA-Tailing Module	261.9	24	10.9		
		Mag-Bind® Total Pure NGS (5 ml)	112.0	50	2.2		

* Illumina sequencing results were typically received within 2 weeks.
** The cost of sequencing per flow cell was normalized to match the average data output of Illumina sequencing, which was 5.8 billion nucleotides. Given that the output of nanopore flow cell was 7.4 billion nucleotides, the nanopore cost was scaled by a factor of 0.784. The entire flow cell was used just for one sample (no multiplexing), as we need as much data as possible.
